# Where do we stand with revision surgery for recurrent carpal tunnel syndrome? A systematic review^☆^

**DOI:** 10.1016/j.jpra.2026.07.039

**Published:** 2026-08-06

**Authors:** Cheuk Ying Kyleen Kiew, Rachel Jia En Lai, David Kwan Ryung Lee, Dave Osinachukwu Duru, Wee Leon Lam

**Affiliations:** a Imperial College London, United Kingdom; b Bart’s and The London School of Medicine and Dentistry, Queen Mary University of London, United Kingdom; c School of Clinical Medicine, University of Cambridge, United Kingdom; d Department of Plastic, Reconstructive and Aesthetic Surgery, Singapore General Hospital, Singapore

**Keywords:** Carpal tunnel, Recurrent, Revision surgery

## Abstract

**Introduction:**

Recurrent carpal tunnel syndrome (CTS) after carpal tunnel release may require revision surgery, but the relative value of decompression, neurolysis, vascularised coverage, nerve wrapping, fat grafting, and other adjuncts remains uncertain. This study synthesised patient-reported outcomes, complications, and re-revision after revision surgery for recurrent CTS, with emphasis on mechanism-based interpretation rather than technique superiority.

**Methods:**

A PRISMA-guided systematic review was performed across PubMed, EMBASE, Scopus, Web of Science and CENTRAL (PROSPERO CRD420261323775). Studies of adults undergoing revision surgery for recurrent CTS and reporting clinical outcomes were included. Owing to heterogeneity in recurrence definitions, operative indications, surgical techniques, follow-up, and outcome reporting, findings were synthesised narratively.

**Results:**

Thirty-five studies (816 patients, 844 hands) were analysed. Most studies were retrospective single-arm case series with heterogeneous definitions, indications, techniques, and outcome measures. Revision surgery was generally associated with symptomatic improvement. Descriptively, pooled VAS improvement was 5.60 points in flap cohorts and 4.08 points in non-flap cohorts, with substantial heterogeneity. Patient-reported outcomes generally improved when reported, but use of validated outcomes was inconsistent. Complications were infrequent. Re-revision was uncommon overall, occurring in 25 cases, representing 3.1% of reported cases, but sparse events and variable follow-up limited interpretation.

**Conclusion:**

Revision surgery for recurrent CTS is generally associated with improved symptoms, but current evidence cannot establish superiority of any technique. Re-revision is best interpreted as an observational durability outcome. Operative planning should be guided by the primary mechanism of failure rather than the presumed superiority of any single revision strategy.

## Introduction

Carpal tunnel syndrome (CTS) is the most common compressive neuropathy of the upper extremity and is typically treated with surgical division of the transverse carpal ligament (TCL) .^1^ Nonetheless, some patients experience persistent, recurrent, or new symptoms after carpal tunnel release (CTR), with reported failure and revision rates varying widely across centres. “Failed CTR” is not a single entity but a heterogeneous spectrum that includes incomplete decompression, secondary sites of compression including double crush or proximal median nerve compression, perineural fibrosis/adhesions, tenosynovitis, systemic neuropathy, and iatrogenic nerve injury, each with distinct diagnostic and therapeutic implications.^2^, ^3^, ^4^, ^5^ As a result, revision surgery is frequently more complex than primary release with more unpredictable outcomes such as residual symptoms despite adequate decompression.^2^^,^^4^

When revision surgery is indicated, options range from re-release and external neurolysis to autologous reconstruction intended to optimise the perineural environment. These include vascularised coverage, most commonly hypothenar fat pad flap (HFPF) or synovial or tenosynovial flap, regional adipofascial flaps, autologous vein wrapping, autologous fat grafting, and commercial nerve wraps.^3^, ^4^, ^5^, ^6^ Soft-tissue coverage provides a gliding interface, reduces re-adhesion and circumferential scar, mechanically cushions the nerve, and for vascularised flaps, improves local microenvironment and neovascular support to the median nerve.^5^^,^^6^ However, despite such benefits, consensus on when coverage adds value and which technique should be preferred remains limited by small numbers, variable definitions of “recurrent” versus “persistent,” and inconsistent outcome reporting.

The existing literature suggests that revision surgery can improve symptoms, but the quality and interpretability of the evidence are limited. Most included studies in existing reviews are small retrospective case series, often with mixed populations of persistent and recurrent symptoms, inconsistent definitions, variable operative techniques, and non-standardised outcomes.^4^^,^^5^^,^^7^^,^^8^ More recent evidence has refined but not resolved this uncertainty. A systematic review focused on autologous nerve coverage identified eight coverage techniques and found generally high rates of symptomatic improvement across approaches, with hypothenar fat pad flap appearing more favourable than synovial flap for some reported outcomes; however, the authors concluded that heterogeneity in case mix, follow-up, and outcome definitions precluded firm conclusions regarding superiority of any technique.^6^ A subsequent meta-analysis similarly suggested potentially favourable results for hypothenar fat pad flap and proposed patient- and biology-related predictors of success, but it remained limited by non-comparative evidence, study-level inference, and inclusion of both recurrent and persistent CTS.^9^ Instructional and state-of-the-art reviews likewise stress that convincing comparative evidence for flap superiority is lacking and that diagnosis, phenotyping, and identification of proximal or alternative pathology are central to management.^4^^,^^10^

Accordingly, the principal gap in the literature is the absence of a synthesis that is explicitly aligned with what the evidence can and cannot support. Existing reviews have largely emphasised symptom improvement across techniques, yet patient-reported improvement after revision surgery is highly vulnerable to confounding by indication, differences in failure phenotype, baseline severity, concomitant procedures, and inconsistent follow-up. By contrast, re-revision is a less frequently reported but clinically meaningful endpoint that may better reflect durability of the revision strategy, even if it remains observational and influenced by local thresholds for reoperation. In addition, the potential role of proximal compression and other failure mechanisms as contributors to poor outcomes has not been adequately integrated into technique-focused evidence synthesis.^4^^,^^5^^,^^10^

Therefore, this systematic review focuses on revision surgery for recurrent CTS with two aims. First, to synthesise outcomes after revision surgery with particular emphasis on patient-reported improvement, complications, and re-revision as an indicator of durability rather than proof of comparative efficacy. Second, to examine whether study-level phenotypic factors, particularly proximal compression, may help explain adverse outcomes and heterogeneity in the published literature. By restricting the review to recurrent CTS and by framing re-revision cautiously as an observational durability outcome, this study seeks to address a more specific and clinically relevant gap than prior broader reviews.

## Methods

### Protocol and registration

This systematic review adhered to the Preferred Reporting Items for Systematic Reviews and Meta-Analyses (PRISMA 2020) guidelines.^11^ The review protocol was prospectively registered on PROSPERO (CRD420261323775).

### Literature search

A comprehensive electronic search of PubMed, EMBASE, Scopus, Web of Science and CENTRAL was conducted from database inception to December 2025. Search terms combined relevant medical subject headings (MeSH) and free-text terms for “recurrent carpal tunnel syndrome,” “revision carpal tunnel release,” “median nerve,” “hypothenar fat pad flap,” “nerve wrap,” “fat graft,” “flap coverage,” and “outcomes.” Boolean operators (AND/OR) structured the query, and reference lists of eligible studies were manually screened to capture additional records.

The initial search yielded 2180 records, reduced to 1238 unique studies after manual de-duplication. Title and abstract screening identified 72 studies, of which 35 met full-text inclusion criteria. See Fig. 1 for PRISMA flowchart.

**Fig. 1:**
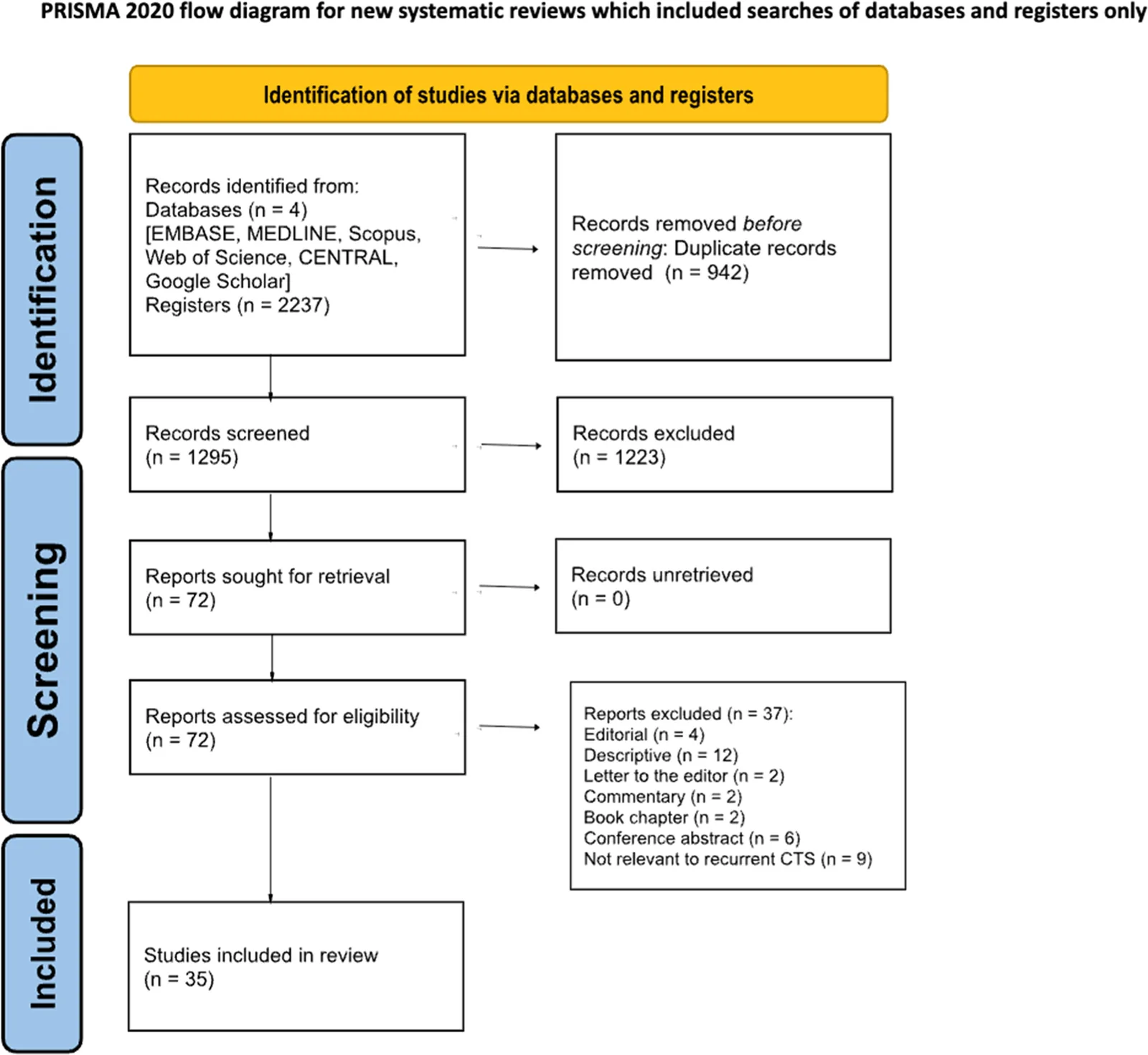
PRISMA flowchart.

### Study selection and eligibility criteria

Two reviewers (CYKK and RL) independently screened all citations using Rayyan. Studies were deemed eligible if they investigated recurrent carpal tunnel release in adults aged 18 years or older and reported at least one relevant clinical outcome such as pain, complications, or re‑revision. Eligible studies were original human research articles published in English up to December 2025 that examined surgical management of recurrent carpal tunnel syndrome, including simple decompression, neurolysis, soft‑tissue or vascularised flap coverage, autologous fat grafting, and biologic or synthetic nerve wraps. Excluded were editorials, reviews, case reports, case series with <3 patients, cadaveric or experimental studies, non‑English publications, and primary carpal tunnel release studies that did not address recurrent pathology. Any discrepancies between reviewers (CYKK and RL) were resolved through discussion, and when consensus could not be reached, a third reviewer (DL) was consulted.

### Data extraction

Full-text data were extracted independently by two reviewers (CYKK, RL). Extracted variables included study design, year, sample size, patient demographics, surgical technique, follow-up duration, pain outcomes (VAS pre/post), functional outcomes (BCTQ SSS/FSS, QuickDASH), complication and re-revision rates, and presence of vascularised coverage or adjunctive grafting. Extracted study-level clinical outcomes, complication events, re-revision events, sensory symptoms, and diagnostic work-up variables were tabulated in the supplementary tables (Tables S1-S5).

### Quality assessment and risk of bias

Risk of bias was assessed according to study design using validated appraisal tools by two reviewers (CYKK, RL). Comparative non-randomised studies was evaluated using ROBINS-I (Risk Of Bias In Non-randomised Studies of Interventions).^12^ Single-arm case series was appraised using the NIH Quality Assessment Tool for Case Series Studies, while purely descriptive case series was assessed using the JBI Critical Appraisal Tool for Case Series.^12^^,^^13^ Disagreements were resolved by consensus. Each study was rated as low, moderate, or serious risk of bias. Detailed ROBINS-I, NIH, and JBI assessments are provided in Tables S6-S8.

### Data synthesis and statistical analysis

Owing to substantial heterogeneity in definitions of recurrence, operative indications, surgical techniques, follow-up duration, outcome measures, and reported denominators, findings were synthesised narratively. Outcomes were grouped by revision strategy and by clinically relevant outcome domain, including pain, validated PROMs, sensory symptoms, electrophysiological findings, complications, and re-revision. Where reported, event counts and denominators were extracted directly and summarised descriptively. No formal meta-analysis or meta-regression was undertaken because the available data were predominantly derived from small non-comparative case series with inconsistent outcome definitions and incomplete reporting of variance measures.

## Results

### Patient demographics and study characteristics

Thirty-five studies and 816 patients (844 hands) undergoing revision surgery for recurrent carpal tunnel syndrome were included (Table 1). The mean patient age at time of revision was 57 years (range 21–92), with a female predominance (M:F = 1:1.67), consistent with the typical epidemiology of primary CTS. Laterality data were reported in 28 studies (n = 651), demonstrating that 66 % (n = 430) of cases involved the dominant or right hand, while bilateral recurrence occurred in approximately 10 % (n = 65) of patients. The mean time period between primary release and revision surgery was 55 months (range 9–165), and the average follow-up duration across studies was 27 months (range: 1.5–132). Most series were Level IV retrospective single-arm case series (80 %, n = 28), reflecting the predominance of single-centre studies. Studies were conducted across Europe (46 %, *n* = 16), North America (46 %, *n* = 16) and Asia/Oceania (9 %, *n* = 3). Reporting of systemic comorbidities, occupational factors, and symptom-duration data were inconsistent. Definitions of recurrence, minimum symptom-free interval after primary release, baseline severity, follow-up duration, and outcome denominators varied substantially across studies.

**Table 1:** Study characteristics and patient demographics.

Study	Design / Country	Revision strategy	Hands / patients	Age, y	Sex, M:F	Time to revision, mo	Follow-up, mo
*Vascularised flap coverage: other vascularised flaps*							
Tham et al., 1996	Prospective series / Australia	Reverse-flow RAFFF	6 / 6	56 (38–76)	1:5	25 (12–1)	24
Mahmoud et al., 2013	Prospective series / USA-Egypt	Other vascularised flap, NR	8 / 8	39 (28–50)	2:6	40 (23–63)	20 (6–38)
Ratigan et al., 2024	Retrospective series / USA	Antebrachial fascia flap + endoscopic TCL release	5 / 4	68 (55–76)	1:3	108	1.5
*Vascularised flap coverage: hypothenar fat pad flap*							
Craft et al., 2007	Retrospective series / USA	CN + HTFPF	28 / 28	68.5 (43–89)	NR	82(5–298)	10.5(3–48)
Fusetti et al., 2009	Prospective series / Switzerland-France	EN + HTFPF	20 / 20	56 (38–85)	8:12	17 (6–42)	13 (12–24)
Karthik et al., 2012	Retrospective analysis / UK	HTFPF	27 / 25	58 (43–81)	10:15	56 (5–262)	22 (10–62)
Jones et al., 2012	Retrospective cohort / USA	EN ± epineurectomy; HTFPF or RFAP subset	18 / 18	55	7:11	21 (7–96)	12
Zieske et al., 2013	Retrospective cohort / USA	EN ± HTFPF or AVW	19 / 18	57	6:13	30 (12–96)	14 (7–23)
Wichelhaus et al., 2015	Retrospective series / Germany	EN + HTFPF	18 / 18	61 (45–71)	4:14	7.5 (5–22)	22 (6–53)
Lattré et al., 2016	Prospective study / Belgium	Pedicled HTFPF	8 / 8	46 (35–55)	2:6	24	12 (3–12)
Athlani and Haloua, 2017	Prospective single-centre / France	EN + pedicled HTFPF	34 / 34	67 (36–91)	19:15	61 (5–240)	24 (12–60)
Pace et al., 2018	Retrospective comparative / USA	OD versus OD + HTFPF	23 / 22	55	9:20	74	53
Sun et al., 2020	Prospective series / USA	EN ± HTFPF	10 / 10	51 (29–71)	3:7	28 (7–52)	12
Ten Heggeler et al., 2022	Registry cohort / Netherlands	Revision CTR, flap use NR	63 / 63	56	1:3	17 (7–38)	6
Bollmann et al., 2023	Retrospective cohort / Switzerland	CN ± HTFPF	36 / 33	56.6 (32–92)	12:21	121	11 (6–30)
Gmainer et al., 2023	Prospective comparative / Austria	EN ± HTFPF	18 / 18	58 (46–70)	8:10	25 (5–63)	18 (10–26)
Akrivos et al., 2026	Retrospective case-control / Greece	OD ± HTFPF	30 / 30	59 (31–81)	9:21	45 (39–50)	132
*Vascularised flap coverage: synovial flap*							
Payr et al., 2019	Prospective study / Austria	EN ± SF ± Z-plasty/STSG	15 / 14	48.5 (22–77)	3:11	20 (19–29)	12
Kimura et al., 2025	Retrospective observational / Japan	SF	10 / 10	73.7 (67–84)	1:9	11.9 (7–24)	44.8 (13–74)
Travis et al., 2026	Retrospective series + survey / USA	CN + SF	17 / 14	64 (43–78)	6:8	165	40
*Non-flap revision: neurolysis/decompression*							
Duclos and Sokolow, 1998	Prospective series / Canada-France	EN	13 / 13	57 (34–85)	NR	20 (3–60)	27 (4–74)
Stirling et al., 2020	Prospective cohort / UK	EN	14 / 13	57 (35–82)	3:10	159	20 (6–69)
Luangjarmekorn et al., 2016	Retrospective comparative / Thailand-USA	EN ± PTR	25 / 22	53 (25–85)	4:18	50 (38–62)	6
*Non-flap revision: autologous vein wrap*							
Varitimidis et al., 2000	Case series / USA	EN + AVW	15 / 15	52 (28–75)	6:9	NR	43 (24–78)
Mulgrew et al., 2012	Retrospective series / UK	EN + AVW	15 / 15	58 (34–90)	9:11	NR	37 (24–50)
*Non-flap revision: implant/commercial barrier*							
Bilasy et al., 2012	Retrospective series / France	EN + Canaletto implant interposition	21 / 20	54 (29–82)	5:15	26	12 (7–19)
Hones et al., 2023	Retrospective series / USA	CN + VersaWrap hydrogel	5 / 5	43 (21–69)	2:3	24 (9–72)	4.6
Non-flap revision: tendon interposition							
Sun et al., 2020	Retrospective comparative / USA	CN + palmaris longus tendon interposition	49 / 49	56.4 (44.4–68.4)	13:36	29 (18–40)	6
Non-flap revision: flexor tenosynovectomy							
Kluemper et al., 2021	Retrospective series / USA	TCL release + FTS	108 / 100	57.5 (33–80)	35:6	120	81
Non-flap revision: autologous fat transfer							
Krześniak and Noszczyk, 2015	Prospective pilot / Poland	AFT	5 / 5	63 (55–76)	2:3	18 (11–36)	14 (12–19)
Cabrejo et al., 2023	Retrospective case-control / USA	AFT	81 / 81	56	1:1	75	17
Gostelie et al., 2026	Prospective multicentre / Netherlands	EN + AFT	29 / 26	55 (35–77)	5:21	NR	63.6
*Mixed or other non-flap revision*							
Cheung et al., 2017	Retrospective case series / USA	EN + pedicled ADMF	14 / 12	64 (49–83)	5:7	NR	44 (12–170)
Eroğlu et al., 2018	Retrospective series / Turkey	EN + TCL release	23 / 23	46.5 (21–69)	8:15	9	8.6 (7–13)

Abbreviations: NR, not reported; RAFFF, radial artery forearm fascial flap; EN, external neurolysis; CN, complete neurolysis; HTFPF, hypothenar fat pad flap; RFAP, radial forearm adipofascial flap; AVW, autologous vein wrap; AFT, autologous fat transfer; PTR, pronator teres release; ADMF, abductor digiti minimi flap; TCL, transverse carpal ligament; OD, open decompression; FTS, flexor tenosynovectomy; SF, synovial flap; STSG, split-thickness skin graft; CTR, carpal tunnel release.

### Surgical techniques and reported indications

Revision surgery most commonly involved repeat open decompression of the carpal tunnel, complete release of recurrent transverse carpal ligament constriction, and external neurolysis of the median nerve. Adjunctive procedures varied according to the mechanism of recurrence. In cases attributed to dense perineural fibrosis and scar tethering, authors frequently used vascularised tissue coverage to recreate a gliding layer between the median nerve and overlying scar. The most commonly reported vascularised coverage technique was the hypothenar fat pad flap (n = 142), followed by vascularised synovial flap coverage (n = 75). Less frequently reported regional options included radial forearm fascial or fasciocutaneous flaps.

Non-vascularised and non-flap adjuncts were heterogenous and included autologous vein wrapping (n = 35), palmaris longus tendon interposition (n = 10), synthetic or implantable barrier devices, autologous fat grafting (n = 107), and flexor tenosynovectomy (n = 108). In some patients, proximal decompression, including pronator release (n = 25), was performed when proximal median nerve compression or double-crush pathology was suspected. Reported mechanisms of recurrence, intraoperative findings, and revision procedures are summarised in Table 2.

**Table 2:** Mechanism of recurrence and revision strategy.

Study	Hands	Phenotype / work-up	Primary failure mechanism	Revision strategy	Technique
*Vascularised flap coverage: HFPF-based*					
Craft et al., 2007	28	Recurrent CTS; NCS/EMG	Dense perineural scarring; tethered median nerve	Re-release, external microneurolysis, HTFPF	Vascularised HFPF
Fusetti et al., 2009	20	Recurrent/recalcitrant CTS; selective ENMG	Perineural fibrosis	Re-release, neurolysis, HTFPF	Vascularised HFPF
Karthik et al., 2012	27	True recurrent CTS; NCS/EMG	Median nerve adherent in scar	Revision decompression, external neurolysis, HTFPF	Vascularised HFPF
Wichelhaus et al., 2015	18	Recurrent CTS; NCS/EMG	Perineural scarring; impaired nerve gliding	Revision decompression, neurolysis, HTFPF	Vascularised HFPF
Lattré et al., 2016	8	Recurrent CTS; NCS/EMG	Scarred revision bed / scarred median nerve	Re-release, limited neurolysis, HTFPF	Vascularised HFPF
Athlani and Haloua, 2017	34	Recurrent CTS / neuropathic pain; NCS/EMG	Perineural scarring and recurrent compression	Revision decompression, neurolysis, HTFPF	Vascularised HFPF
Sun et al., 2020	10	Recurrent CTS; NCS/EMG	Perineural fibrosis	Re-release, neurolysis ± HTFPF	Selective HFPF
Ten Heggeler et al., 2022	63	Recurrent subset; non-standardised work-up	Recurrent compression; dense scar in selected cases	Revision decompression ± neurolysis ± HTFPF	Mixed / selective HFPF
Gmainer et al., 2023	18	Recurrent CTS; NCS/EMG	Dense scar around median nerve	Revision decompression, neurolysis ± fat flap	Selective HFPF
*Vascularised flap coverage: synovial flap*					
Payr et al., 2019	15	True recurrent CTS; NCS/EMG	Scarred median nerve; deficient soft-tissue envelope	Neurolysis ± epineurotomy, SF ± Z-plasty/STSG	Vascularised synovial flap
Bollmann et al., 2023	36	True recurrent CTS; NCS/EMG	Epineural fibrosis; poor gliding bed	Revision decompression, neurolysis, SF	Vascularised synovial flap
Kimura et al., 2025	10	Recurrent CTS; NCS/EMG	Median nerve adhesion after prior CTR	External neurolysis, flexor synovial flap	Vascularised synovial flap
Travis et al., 2026	17	Recurrent CTS after ≥12-month symptom-free interval; NR work-up	Recurrent scar around median nerve	Revision decompression, complete neurolysis, SF	Vascularised synovial flap
*Vascularised flap coverage: other flaps*					
Tham et al., 1996	6	Multiply recurrent CTS; NCS/EMG	Chronically scarred, adherent median nerve	Neurolysis, reverse-flow radial artery fascial flap	Vascularised fascial flap
Jones et al., 2012	18	Failed CTR cohort with recurrent subset; selective NCS/EMG	Fibrosis, tethering, traction neuritis; incomplete/recurrent compression	Neurolysis ± epineurectomy ± HFPF/RFAP	Mixed / selective flap
Mahmoud et al., 2013	8	Recurrent CTS; NCS/EMG	Scarred median nerve requiring vascularised gliding bed	Neurolysis ± epineurotomy, radial forearm fascial flap	Vascularised fascial flap
Cheung et al., 2017	14	Recurrent CTS ± painful scar/CRPS; NCS/EMG	Painful scar; scarred median nerve	Neurolysis, pedicled ADMF	Vascularised muscle flap
Ratigan et al., 2024	5	Recurrent CTS after open CTR; NCS/EMG	Residual/recurrent TCL compression	Endoscopic revision release using antebrachial fascial/local fascial approach	Endoscopic decompression
*Non-flap revision: decompression / neurolysis*					
Duclos and Sokolow, 1998	13	True recurrent CTS; selective NCS/EMG	Perineural scarring after complete release	Revision decompression, external neurolysis ± tenosynovectomy	Neurolysis alone
Eroğlu et al., 2018	23	Recurrent CTS; NCS/EMG	Incomplete TCL release	Release of residual TCL	Decompression alone
Stirling et al., 2020	14	True recurrent CTS; NCS/EMG	Recurrent compression / scarred retinaculum	Revision decompression and external neurolysis	Neurolysis alone
Luangjarmekorn et al., 2016	25	Failed CTR with suspected proximal compression; NCS/EMG	Wrist ± pronator-level compression	Revision CTR ± proximal median nerve release	Dual-site decompression
*Non-flap revision: barrier, interposition, or grafting*					
Varitimidis et al., 2000	15	Recurrent compressive neuropathy; NCS/EMG	Recurrent perineural fibrosis	Neurolysis, saphenous vein wrap	Autologous vein wrap
Mulgrew et al., 2012	15	Recalcitrant recurrent neuropathy; NCS/EMG	Nerve embedded in recurrent scar	Neurolysis, autologous vein wrap	Autologous vein wrap
Bilasy et al., 2012	21	Recurrent CTS; NCS/EMG	Perineural fibrosis / neoretinaculum	Neurolysis, Canaletto implant interposition	Commercial barrier
Sun et al., 2020	49	Recurrent CTS; NCS/EMG	Perineural fibrosis	Complete neurolysis, palmaris longus tendon interposition	Tendon interposition
Hones et al., 2023	5	Recurrent compressive neuropathy; NR work-up	Recurrent scarring after prior release	Complete neurolysis, VersaWrap hydrogel protector	Commercial barrier
Cabrejo et al., 2023	81	Recurrent CTS; NCS/EMG	Scarred revision bed; transection/neuroma excluded	Revision decompression with AFT	Fat grafting
Gostelie et al., 2026	29	Recalcitrant CTS after ≥2 CTRs; NCS/EMG	Dense perineural adhesions after failed neurolysis	Extensive neurolysis with lipoaspirate/fat grafting	Fat grafting
*Non-flap revision: synovial debulking*					
Kluemper et al., 2021	108	Recurrent idiopathic CTS; variable NCS/EMG	Hypertrophic fibrotic flexor tenosynovium	Revision decompression with flexor tenosynovectomy	Tenosynovectomy
Comparative or mixed cohorts					
Pace et al., 2018	23	Recurrent and persistent CTS; inconsistent work-up	Scarring, fibrosis, tenosynovitis, recurrent compression	Revision decompression ± HTFPF	Mixed / comparative
Akrivos et al., 2026	30	Recurrent CTS after symptom-free interval; NCS/EMG	Scarred median nerve / recurrent compression	Revision decompression ± HTFPF	Mixed / comparative HFPF
Zieske et al., 2013	19	Recurrent CTS subgroup; NCS/EMG	Fibrosis, tethering, perineural adhesions	Revision decompression, neurolysis ± HTFPF/AVW	Mixed / comparative

Abbreviations: ADMF, abductor digiti minimi flap; AFT, autologous fat transfer; AVW, autologous vein wrap; CRPS, complex regional pain syndrome; CTR, carpal tunnel release; CTS, carpal tunnel syndrome; EMG, electromyography; ENMG, electroneuromyography; HFPF, hypothenar fat pad flap; NCS, nerve conduction studies; NR, not reported; RFAP, radial forearm adipofascial flap; SF, synovial flap; STSG, split-thickness skin graft; TCL, transverse carpal ligament.

### Pain improvement (VAS)

Pain outcomes were reported using heterogeneous measures, most commonly VAS pain scores, pain-free status, or subjective pain improvement, with variable timing of postoperative assessment. In vascularised flap cohorts, reductions in VAS were observed after external neurolysis with hypothenar fat pad flap (6.1 to 1.5), pedicled hypothenar fat pad flap (6.4 to 1.4), external neurolysis with or without hypothenar fat pad flap (8.2 to 2.1), and synovial flap reconstruction (8.5 to 2.2). Pain-free or improved pain outcomes were also frequently reported after flap coverage.

Non-flap revision strategies also demonstrated pain improvement. Reductions in VAS were reported after autologous vein wrapping (7.5 to 4.0), external neurolysis with vein wrapping (7.0 to 2.0), implant interposition (6.45 to 3.86), palmaris longus tendon interposition (6.2 to 2.8), and open decompression (8.0 to 1.8). Larger non-flap cohorts observed pain improvements after flexor tenosynovectomy (104, 96%), palmaris longus interposition (45, 92%), and external neurolysis with autologous fat transfer (25, 86%), where reported.

Revision surgery was generally associated with clinically meaningful pain reduction. However, the magnitude of improvement varied widely between cohorts, and interpretation was limited by differences in baseline severity, operative indication, adjunctive procedures, and follow-up duration.

Reported VAS improvement appeared broadly favourable across both flap and non-flap cohorts; however, differences between technique categories cannot be interpreted comparatively because of baseline heterogeneity, confounding by indication, and inconsistent follow-up. Summary outcomes by revision technique are provided in Table 3, with study-level clinical outcome details provided in Table S1.

**Table 3:** Summary of reported outcomes by revision technique.

Revision technique	Studies / hands	Typical indication / rationale	Reported clinical benefit	Electrodiagnostic findings	Complications / re-revision	Key limitation
Repeat decompression / external neurolysis alone	3 / 50	Residual or re-formed TCL; recurrent compression or perineural scar without planned interposition	Pain improved in 46/50 hands; QuickDASH improved where reported; Tinel’s sign commonly resolved; improved nerve glide/ROM described	NCS improved or normalised in most tested hands; latency normalisation reported in 21/23 hands in one series	3 / 0	Small uncontrolled series; mixed mechanisms, especially incomplete release versus scar-mediated recurrence
Hypothenar fat pad flap	10 / 262	Scar-mediated recurrent CTS with perineural fibrosis, tethered median nerve, poor glide, or poor soft-tissue bed	Consistent pain improvement; pain-free status in 66/83 hands where specified; QuickDASH/BCTQ improved where measured; improved nocturnal symptoms, paresthesia, Tinel’s sign, glide, and function widely reported	NCS/EMG generally improved when repeated, including latency or conduction velocity; testing not standardised	9 / 11	Largest evidence base, but mostly uncontrolled case series; HFPF nearly always combined with decompression/neurolysis, limiting attribution to flap
Synovial flap	3 / 42	Scarred median nerve requiring vascularised gliding tissue; sometimes deficient local envelope or skin shortage	VAS and QuickDASH improved where reported; Tinel’s sign improved/resolved; early ROM recovery described; all 10 hands improved in the most recent small series	Distal motor latency and nerve conduction improved in studies repeating testing	3 / 0	Sparse technique-specific evidence; small series and heterogeneous adjunctive skin procedures
Other vascularised flaps / adipofascial coverage	5 / 51	Severe or multiply operated scarred tunnel, painful scar, deficient soft-tissue envelope, or need for vascularised coverage beyond HFPF	Pain improved in 44/51 hands; sensory improvement, Tinel’s resolution, and ROM recovery commonly reported	NCS improved in most studies reporting postoperative testing, including latency and conduction velocity	9 / 8	Heterogeneous procedures and indications; re-revision burden likely reflects severe mixed cohorts
Autologous vein wrapping	2 / 30	Multiply operated recurrent perineural fibrosis; biologic barrier after neurolysis	VAS improved from approximately 7–7.5 to 2–4; pain improved in 30/30 hands; Tinel’s sign and sensory discrimination improved in most reported hands	Sensory and motor latency improved; sensory velocity improved where reported	7 / 1	Very small evidence base; donor-site morbidity and wrap-related complications inconsistently defined
Synthetic / biologic nerve wrap or implant	2 / 26	Recurrent scarring after prior release; commercial barrier protection after neurolysis	Pain improved in most hands; QuickDASH improved in implant series; subjective improvement in all hydrogel cases; Tinel’s sign improved where measured	NCV improved in implant series; postoperative NCS not reported for hydrogel wrap	7 / 1	Small product-specific series using different materials; durability and complication profile uncertain
Autologous fat grafting / fat transfer	2 / 110	Scar modulation and soft-tissue padding after decompression or extensive neurolysis, often in recalcitrant or multiply operated CTS	Clinical improvement in 25/29 hands in one series and 92% in another; Tinel’s sign resolved in larger series; improved soft-tissue glide and unrestricted motion described	Postoperative improvement incompletely reported; one series showed improved NCS index and return of recordable responses in several hands	2 / 0	Promising but heterogeneous; used variably as adjunctive scar modulation rather than standalone revision
Flexor tenosynovectomy / debulking adjunct	1 / 108	Hypertrophic fibrotic flexor tenosynovium causing recurrent idiopathic CTS	Pain improved in 104/108 hands; postoperative QuickDASH 31.2; symptom improvement common, but sensory outcomes not granular	NCS severity improved, but quantitative values not provided	5 / 0	Single retrospective series; most applicable to tenosynovial hypertrophy rather than scar-tethered median nerve recurrence
Palmaris longus / tendon interposition	1 / 49	Perineural fibrosis; tendon graft used as interposition barrier after decompression/neurolysis	VAS improved from 6.2 to 2.8; pain improved in 45/49 hands; Tinel’s sign decreased from 27/49 to 7/49 hands	Distal motor latency improved by approximately 0.9 ms	1 / 2	Single series; inconsistent BCTQ reporting; mechanism differs from vascularised coverage
Combined wrist and proximal decompression	1 / 25	Failed CTR with suspected concomitant proximal median nerve compression, including pronator-level pathology	Pain improved in 14/25 hands; Tinel’s sign decreased from 25/25 to 2/25 hands; ROM normal	NCS/EMG qualitatively improved	0 / 0	Applicable only when proximal compression is clinically suspected; not a strategy for isolated scar-mediated recurrence
Mixed techniques / unclear comparative cohorts	3 / 56	Heterogeneous real-world revision cohorts using decompression ± HFPF, vein wrap, or other adjuncts based on operative findings	Pain improved in 45/56 hands; VAS/QuickDASH improved where reported; Tinel’s sign and strength improved in selected cohorts	NCS improved in some cohorts, but reporting was incomplete or study-level	1 / 2	Useful for practice-pattern context, but not suitable for technique-specific inference

Values are crude technique-category summaries from included clinical studies and should not be interpreted as pooled comparative estimates.

Abbreviations: BCTQ, Boston Carpal Tunnel Questionnaire; CTS, carpal tunnel syndrome; CTR, carpal tunnel release; EMG, electromyography; HFPF, hypothenar fat pad flap; NCS, nerve conduction studies; NCV, nerve conduction velocity; PROM, patient-reported outcome measure; QuickDASH, shortened Disabilities of the Arm, Shoulder and Hand questionnaire; ROM, range of motion; TCL, transverse carpal ligament; VAS, visual analogue scale.

### Patient-reported outcome measures

Validated patient-reported outcome measures, including the Boston Carpal Tunnel Questionnaire (BCTQ) Symptom Severity Scale (SSS) and Functional Status Scale (FSS), and QuickDASH were inconsistently used. Where reported, PROMs generally improved after revision surgery. Improvements in QuickDASH were reported in hypothenar fat pad flap coverage (68.2 to 20.7), synovial flap reconstruction (52.4 to 19.3), and open decompression (65 to 18.5), exceeding the MCID of 18 points^14^ Improvements in BCTQ were reported less frequently but included SSS (3.05 to 1.09) and FSS (2.71 to 1.16) after pedicled hypothenar fat pad flap coverage, and SSS (2.7 to 1.92) after external neurolysis with or without hypothenar fat pad flap. Where paired BCTQ data were available, reporrted changes exceeded the proposed minimal clinically important difference (MCID) for SSS/FSS of 1.05/1.13.^15^ PROM reporting was limited by small sample sizes, inconsistent reporting of paired preoperative and postoperative data, variable follow-up, and substantial heterogeneity in revision indications. Technique-level PROM summaries are shown in Table 3, and study-level PROM data are provided in Table S1.

### CTS-specific sensory symptoms

Several studies reported CTS-specific sensory symptoms including numbness, paresthesia, and nocturnal awakening. However, such outcomes were inconsistently defined and were usually reported as residual postoperative symptoms. Postoperative numbness was observed in 63 (7.5%) of case, paresthesia in 27 (3.2%), and nocturnal awakening in 21 (2.5%) of cases. Persistent nocturnal symptoms were uncommon after revision surgery, although formal pooling was limited by inconsistent definitions, variable follow-up, and incomplete use of disease-specific symptom instruments. Detailed CTS-specific sensory symptom reporting is providede in Table S5.

### Electrophysiological recovery

Electrophysiological outcomes were reported inconsistently and included variable combinations of distal motor latency, sensory latency, sensory conduction velocity, sensory amplitude, and qualitative nerve conduction improvement. Several studies reported postoperative improvement in nerve conduction after revision surgery, including sensory nerve conduction velocity (39.6 to 49.4 m/s) and distal motor latency (4.0 to 3.5 ms) after circumferential neurolysis with or without hypothenar fat pad flap coverage, distal motor latency improvement (5.1 to 3.8 ms) after synovial flap reconstruction, and sensory latency (4.5 to 3.0 ms) and motor latency improvements (5.5 to 3.8 ms) after autologous vein wrapping. Other studies reported qualitative improvement, including normalisation or improvement after external neurolysis with hypothenar fat pad flap coverage (n = 14, 78%), external neurolysis alone (n = 5, 83%), and latency normalisation after revision release with external neurolysis (n = 21, 91%), where reported. Definitions of electrophysiological recovery were heterogeneous and were not consistently paired with clinical outcomes. Technique-level electrophysiological outcomes are summarised in Table 3, with study-level electrodiagnostic findings detailed in Table S1.

### Complications

Postoperative complications were low (5.7%, n = 47), where reported (n = 824), and ranged from minor wound complications and scar symptoms to infection, complex regional pain syndrome (CRPS), implant-related events, and donor-site morbidity principally in vein-harvest cohorts. Flap loss was not reported in the vascularised flap cohorts. Summary complication rates by revision technique are shown in Table 3, with study-level complication details provided in Table S2.

### Re-revision

Re-revision was uncommon. Re-revision rate was low (n = 25, 3.1%), with the most reported in HFPF cohorts (n = 11, 14%) and in patients receiving external neurolysis with or without epineurectomy (n = 8, 44%), followed by isolated events after autologous vein wrapping, implant interposition, tendon interposition, open decompression, and mixed neurolysis/wrapping procedures. Summary re-revision rates by revision technique are shown in Table 3, with study-level re-revision events and denominators detailed in Table S3.

### Proximal compression and mechanism of recurrence

Proximal median nerve compression or double-crush pathology was inconsistently reported. Where reported, proximal compression was generally treated as a distinct contributor to persistent or recurrent symptoms rather than as direct evidence of failure of the index carpal tunnel release. A small number of studies incorporated proximal decompression, most commonly pronator release, in addition to revision carpal tunnel surgery. However, such cohorts are clinically heterogenous and available data were insufficient to determine whether proximal compression independently influenced pain relief, complications, or re-revision. Reported mechanisms of recurrence and intraoperative findings are summarised in Table 2, while study-level diagnostic work-up and proximal compression reporting are summarised in Table S4.

### Quality of evidence and risk of bias

Risk of bias was assessed using three different tools appropriate to study design, including the ROBINS-I tool for comparative non-randomised studies, the NIH Quality Assessment Tool for Case Series Studies for single-arm case series, and the JBI Critical Appraisal Tool for Case Series for descriptive studies (Fig. 3). Comparative non-randomised evidence was at moderate to serious risk of bias, driven mainly by confounding factors such as non-random group allocation and limited adjustment for prognostic factors, subjective outcome measurement, and potential selective reporting with a lack of pre-specified protocols. Most single-arm case series were fair-to-moderate quality. Common limitations were predominantly descriptive outcome reporting with minimal statistical analysis, inconsistent or non-validated outcome measures, unclear handling of missing data, and limited assessor standardisation. The descriptive case series showed variable quality, with unclear inclusion criteria, non-consecutive or incomplete case inclusion, and non-standardised outcome assessment, limiting interpretation of findings. Detailed risk-of-bias and quality assessments are provided in Tables S6-S8.

## Discussion

This systematic review synthesised outcomes of revision surgery for recurrent CTS across 816 patients and 844 hands from 35 studies. Revision surgery was generally associated with improvement in pain and function, while sensory recovery and electrophysiological improvement were less consistently reported. However, the evidence base consisted mainly of retrospective, single-arm case series with heterogeneous definitions of recurrence, variable follow-up, inconsistent outcome measures, and mixed adjunctive procedures. Accordingly, these findings should be interpreted as a descriptive synthesis of reported outcomes rather than comparative evidence that flap-based reconstruction is superior to non-flap revision.

VAS pain improvement was numerically greater in flap cohorts than non-flap cohorts, and reported improvements in BCTQ-SSS, BCTQ-FSS, and QuickDASH often exceeded published MCIDs.^14^^,^^15^ These findings are clinically encouraging but should be considered hypothesis-generating. Differences in baseline severity, failure mechanisms, prior surgery, comorbidities, adjunctive procedures, and follow-up timing limit direct comparisons between techniques. PROM change alone is therefore an unstable discriminator of technique performance in the current literature.

Prior reviews have reached conflicting conclusions regarding median nerve coverage in recurrent surgeries. Soltani et al. (2013) reported higher success rates with flap coverage than non-flap revision, whereas de Roo et al. (2021) found no clear added value for coverage, with similar pooled success rates after hypothenar fat pad flap, synovial flap, and repeat decompression alone. More recent reviews of autologous coverage report favourable outcomes for hypothenar fat pad flap and other vascularised techniques, but largely within selected non-comparative cohorts.^6^^,^^9^ Similarly, Akrivos et al. (2026) found improvement after both open decompression and hypothenar fat pad flap, without a statistically significant between-group difference at long-term follow-up. The apparent disagreement across studies likely reflects differences in case definition, failure phenotype, and outcome ascertainment more than the intrinsic superiority of any single technique.

The key clinical issue before revision surgery is not simply whether median neuropathy persists, but why symptoms have returned and whether the cause is surgically correctable at the wrist. Persistent symptoms after CTR suggest incomplete release, incorrect diagnosis, proximal compression, cervical radiculopathy, or systemic neuropathy. Truly recurrent symptoms after a symptom-free interval are more compatible with recurrent compression, perineural fibrosis, scar tethering, or recurrent tenosynovitis. New postoperative symptoms raise concern for iatrogenic nerve injury, neuroma, pillar pain, or CRPS.^2^, ^3^, ^4^, ^5^ This distinction was inconsistently applied across included studies and remains a major limitation of technique-specific interpretation.

A practical pre-revision workup should combine a careful history and examination, a review of the index operation, an assessment of the symptom-free interval, repeat electrodiagnostic testing, and selective imaging. Nerve conduction studies and EMG should confirm recurrent median neuropathy, grade severity, identify denervation, and assess for cervical radiculopathy, polyneuropathy, or proximal median nerve compression. High-resolution ultrasound may help identify incomplete release, residual transverse carpal ligament, focal nerve swelling, impaired nerve glide, tenosynovitis, mass lesions, anomalous anatomy, or perineural fibrosis^16^; it has been reported to show good correlation with operative findings in failed decompression. MRI is not routinely necessary but may be useful when a mass, anomalous anatomy, extensive scarring, or unclear diagnosis is suspected. A wrist-only revision is unlikely to succeed when symptoms are primarily driven by proximal median nerve compression, cervical radiculopathy, systemic neuropathy, or postoperative pain syndromes. A proposed mechanism-based approach to recurrent symptoms after carpal tunnel release is shown in Fig. 2.

**Fig. 2:**
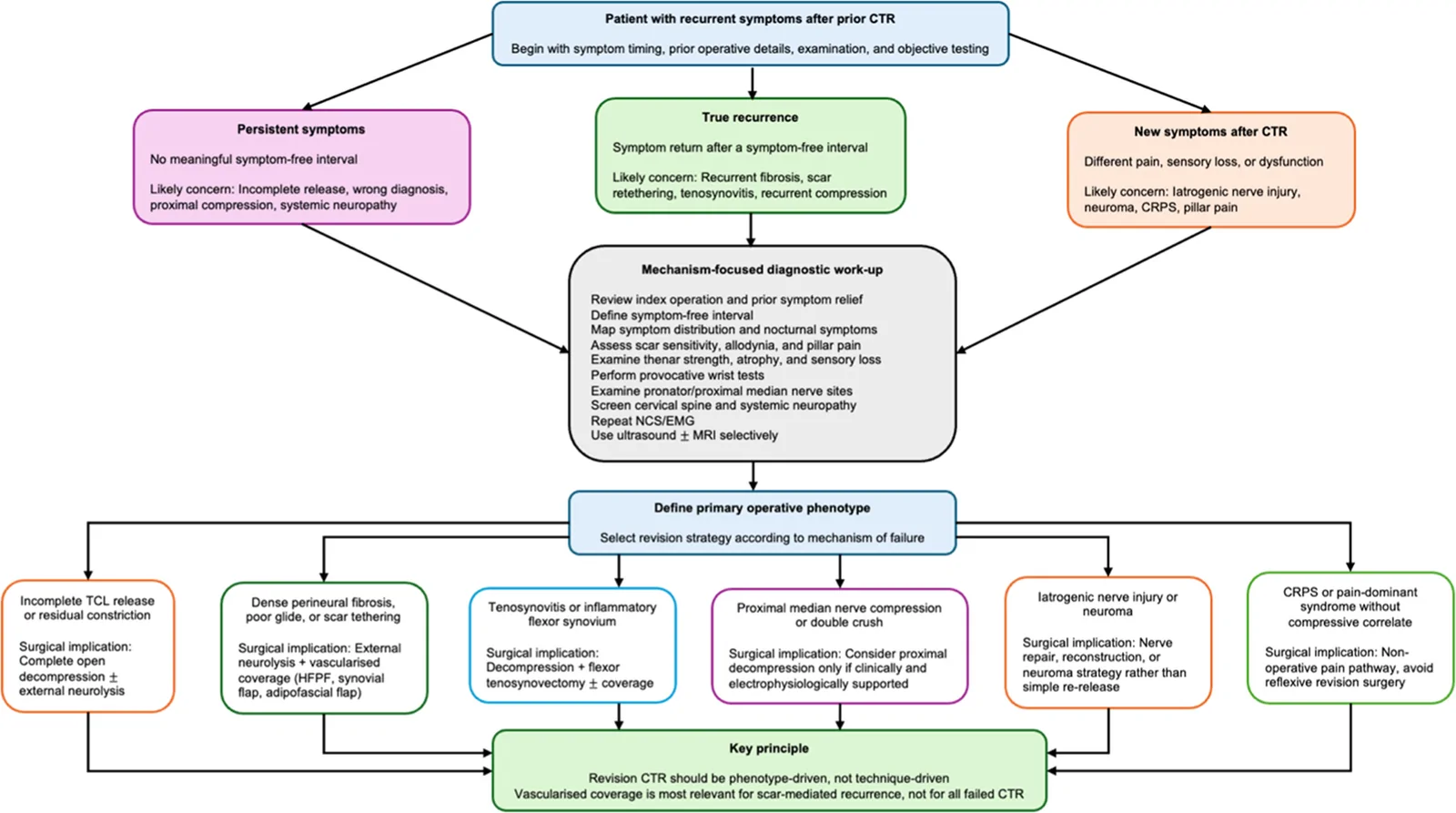
Mechanism-based approach to recurrent symptoms after carpal tunnel release. Patients presenting after prior carpal tunnel release should first be classified according to symptom timing: persistent symptoms, true recurrence after a symptom-free interval, or new symptoms. Diagnostic work-up should define the likely mechanism of failure using operative history, symptom distribution, examination, electrodiagnostic testing, and selective imaging. Revision surgery should then be tailored to the dominant phenotype rather than applied as a uniform re-release strategy. Vascularised coverage is most relevant for scar-mediated recurrence with poor nerve glide or perineural tethering, whereas incomplete release, proximal compression, iatrogenic nerve injury, and pain-dominant syndromes require different treatment pathways.

**Fig. 3:**
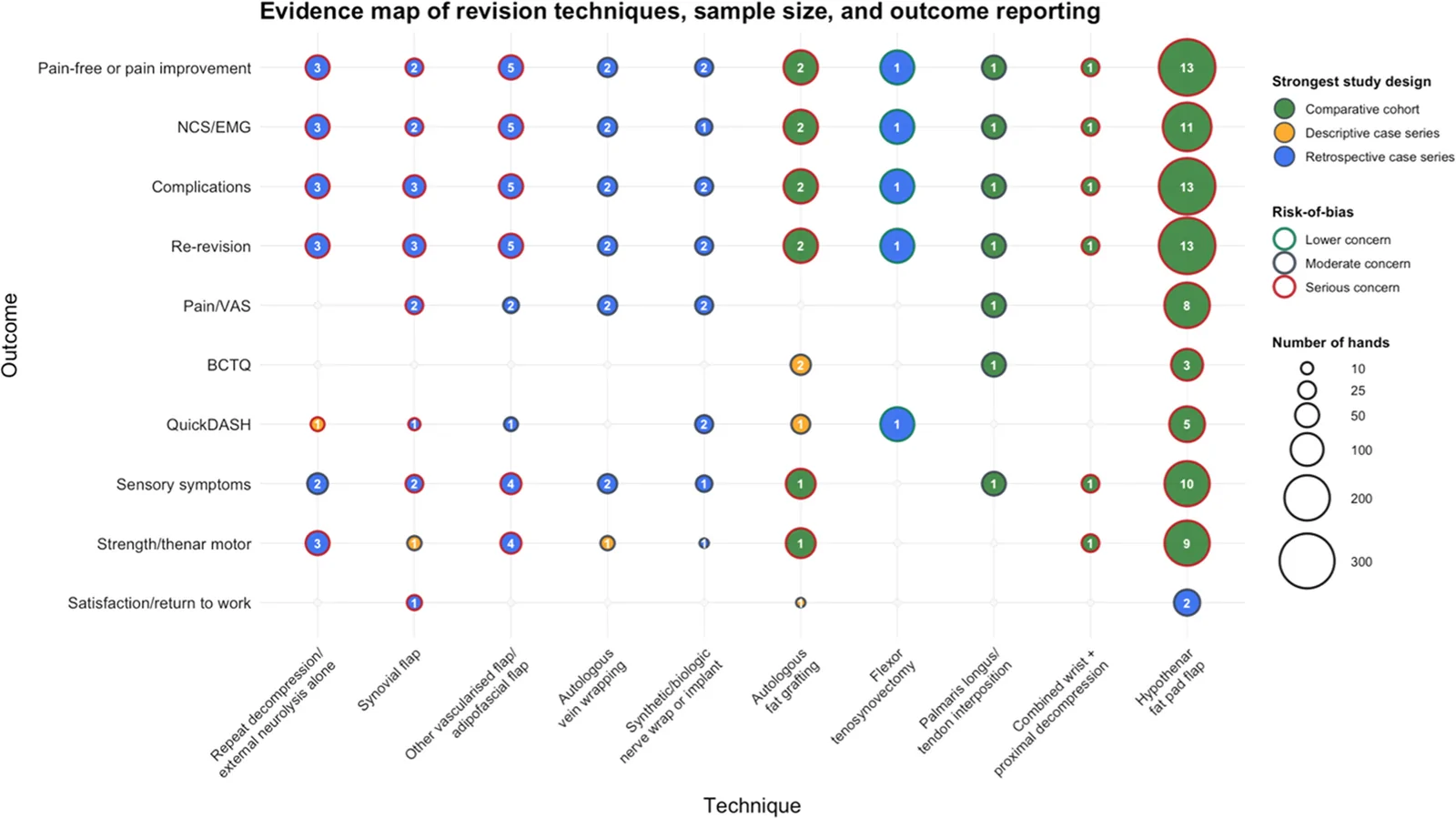
Evidence map of revision techniques and outcome reporting. Bubble size represents the number of hands contributing data for each technique-outcome domain. Colour indicates study design or overall risk of bias. The map demonstrates that most evidence consists of small non-comparative cohorts, with inconsistent reporting of validated PROMs, electrophysiological recovery, complications, and re-revision.

Proximal median nerve compression and double-crush pathology require particular attention because they may limit the response to wrist-only revision regardless of flap coverage.^17^^,^^18^ In this review, proximal compression was inconsistently assessed and only occasionally treated with additional decompression, most commonly pronator release. The available data are therefore insufficient to determine whether proximal compression independently influenced pain relief, complications, or re-revision.

Patient counselling should reflect the lower ceiling of recovery after revision surgery. Westenberg et al. found that patients undergoing revision CTR had higher BCTQ scores, more pain, and lower satisfaction at long-term follow-up than matched patients undergoing a single CTR, and pre-revision thenar atrophy was independently associated with worse pain outcomes.^19^ Thus, revision surgery may improve symptoms without restoring patients to the outcome expected after uncomplicated primary release, particularly when longstanding neuropathy or irreversible denervation is present.

The mean interval between primary release and revision was 55 months, but the range was wide, from 9 to 165 months. Early symptom return may reflect incomplete release, incorrect diagnosis, or unrecognised proximal compression, whereas late recurrence after a symptom-free interval is more consistent with recurrent fibrosis, tenosynovitis, or progressive systemic neuropathy. Future studies should report the symptom-free interval separately from time to revision, as referral delay and patient tolerance may obscure the biological timing of recurrence.

Re-revision is a clinically meaningful durability outcome as it reflects failure severe enough to prompt further surgery. In this review, re-revision was uncommon, but sparse events, variable follow-up, incomplete surveillance, and surgeon or patient thresholds for reoperation limit interpretation. The concentration of re-revision events in cohorts treated with external neurolysis with or without epineurectomy should also be interpreted cautiously because these procedures may have been selected for more severe perineural scarring or recurrent constriction. Nevertheless, it remains biologically plausible that neurolysis alone may not prevent recurrent adhesion when the perineural bed remains scarred.

Vascularised coverage is beneficial in scar-mediated recurrence as it may provide interposition, improve the local soft-tissue bed, and recreate a gliding layer around a nerve tethered by recurrent fibrosis.^20^^,^^21^ However, this rationale should not be generalised to all recurrent or failed CTR presentations. If incomplete release is the main finding, complete decompression is the priority.^20^ Symptoms driven by proximal median nerve compression, cervical radiculopathy, multifocal neuropathy, or iatrogenic nerve injury are unlikely to be corrected by flap coverage alone.

Revision CTS should be approached as a phenotype-driven rather than technique-driven problem. Important phenotypic features include timing of recurrence, residual constriction, degree of perineural fibrosis, quality of the local soft-tissue bed, nerve mobility, baseline nerve injury, and the presence of proximal or systemic neuropathy. Vascularised coverage may be considered when exploration demonstrates dense perineural fibrosis, poor nerve glide, recurrent scar tethering, compromised local soft tissue, dysvascular or hourglass-constricted nerve, or repeated scar bridging. Non-flap revision may be sufficient when residual constriction or incomplete release is present without substantial perineural scarring. Proximal decompression should be reserved for patients with clinical, electrodiagnostic, or imaging evidence of a relevant proximal compression site. Flexor tenosynovectomy may be appropriate when hypertrophic or inflammatory tenosynovium contributes to recurrent compression. Other adjuncts, including fat grafting, vein wrapping, tendon interposition, and synthetic or xenogeneic barriers, may be useful in selected cases, but current evidence is too sparse and heterogeneous to compare them meaningfully with vascularised coverage.

Complication rates were low and broadly similar across flap and non-flap cohorts, and no flap loss was reported. However, complication reporting was inconsistent and likely underestimates minor or late morbidity. Overall complication rates may also obscure differences in morbidity type, including additional dissection for flaps, donor-site morbidity for vein wrapping, and implant-related risks for synthetic or xenogeneic barriers. Patient factors including diabetes, tobacco use, rheumatoid arthritis, concomitant cubital tunnel syndrome, CRPS, longer symptom duration, higher baseline BCTQ scores, and thenar atrophy have been associated with revision risk or worse revision outcomes and should inform counselling.^19^^,^^22^, ^23^, ^24^

This review has several limitations. Most included studies were retrospective, single-arm Level IV case series, with no randomised trials and few comparative studies. Technique-stratified analyses are therefore vulnerable to confounding by indication and cannot establish comparative effectiveness. Definitions of recurrent CTS, minimum symptom-free interval, baseline severity, prior surgery, operative indication, outcome measures, and follow-up intervals varied substantially. Reporting of complications, re-revision, proximal compression, systemic neuropathy, occupational factors, and comorbidities was incomplete. Psychological factors such as depression, pain catastrophising, treatment expectations, and illness perceptions were also not captured, despite evidence that they influence outcomes after CTR (Núñez-Cortés et al., 2022; Sun et al., 2021). Publication bias is also possible, particularly among small single-centre series reporting favourable outcomes for specific techniques.

Future studies should prospectively classify failed CTR by mechanism rather than broad labels such as recurrent or persistent CTS. They should distinguish persistent, recurrent, and new symptoms; report the symptom-free interval; document prior operative findings and number of previous procedures; and record evidence of incomplete release, perineural fibrosis, proximal compression, cervical radiculopathy, systemic neuropathy, tenosynovitis, or iatrogenic nerve injury.^25^^,^^26^ Standardised diagnostic pathways should include repeat electrodiagnostic testing and high-resolution ultrasound, with selective MRI when indicated.^16^ A core outcome set should include VAS pain, BCTQ-SSS/FSS, QuickDASH or another upper-limb disability measure, nocturnal symptoms, objective sensory testing, thenar motor status, electrodiagnostic parameters, complications, satisfaction, return to work, and re-revision. Comparative studies should adjust for baseline severity, comorbidities, mechanism of recurrence, intraoperative scarring, adjunctive procedures, proximal compression, and psychological factors.^24^^,^^27^ Re-revision should be reported as a mandatory durability endpoint with at least 2 years of follow-up, as shorter follow-up may miss delayed recurrent scarring or failure.^19^

## Funding statement

None.

## Ethical approval

Not required.

## Declaration of competing interest

None declared.
