# Supplementary material for: Where do we stand with revision surgery for recurrent carpal tunnel syndrome? A systematic review
Source: JPRAS Open. 2026 Aug 6;52:1–14. doi: 10.1016/j.jpra.2026.07.039 (PMC13731119; doi:10.1016/j.jpra.2026.07.039)
Supplement: Supplementary file 1 [file mmc1.docx]

Table S1. Detailed clinical outcomes, complications, and electrodiagnostic findings.

| Author (Year) | Hand (n) | Patient (n) | Procedure (revision) | Complications (n/N hands) | Re-revision (n/N) | VAS (Pre → Post) | Pain-free (n/N) / Pain improvement | QuickDASH (Pre → Post) | BCTQ SSS (Pre → Post) | BCTQ FSS (Pre → Post) | NCS/EMG outcome (reported metric) | Tinel’s sign (Pre → Post) | ROM / strength |
| --- | --- | --- | --- | --- | --- | --- | --- | --- | --- | --- | --- | --- | --- |
| Vascularised Flap Coverage: HFPF-based (± neurolysis / re-release) |  |  |  |  |  |  |  |  |  |  |  |  |  |
| (Craft et al., 2007) | 28 | 28 | CN + HFPF | NR | 0/28 | NR → 83% no pain | Pain-free 23/28 | NR | NR | NR | All improved (not quantified) | 28/28 → 2/28 | Full gliding restored |
| (Fusetti et al., 2009) | 20 | 20 | EN + HFPF | 2/20 | 0/20 | Severe pain in >90% → ~85% VAS decrease | Pain-free 18/20; pain improvement 18/20 | 75.8 → 18.2 | NR → NR | NR → NR | ENMG improved in 3/4 tested | 20/20 → 3/20 | Grip +13 kg |
| (Karthik et al., 2012) | 27 | 25 | HFPF | 0/27 | 0/27 | NR → NR | Pain-free 24/27; pain improvement 24/27 | NR | NR | NR | NCS abnormal in all; improved postop (qualitative) | 23/27 → 3/27 | Normal |
| (Wichelhaus et al., 2015) | 18 | 18 | EN + HFPF | 2/18 | 0/18 | 6.1 (SD 1.9) → 1.5 (SD 1.9) | Pain improvement 15/18 | 42 (SD 21) → 18 (SD 20) | NR | NR | Velocity/latency normalised in 14/18 | 18/18 → 2/18 | Full restored |
| (Lattré et al., 2016) | 8 | 8 | Pedicled HFPF | 0/8 | 0/8 | NR → 0.38 | Pain improvement 8/8 | NR | 3.05 (SD 0.81) → 1.09 (SD 0.20) | 2.71 (SD 0.84) → 1.16 (SD 0.20) | EMG: compression pre-op; 1 normalised at 1 year | 8/8 → 0/8 | Full ROM by 12 mo |
| (Athlani and Haloua, 2017) | 34 | 34 | EN + pedicled HFPF | 0/34 | 0/34 | 6.4 → 1.4 | Pain improvement 32/34 | 60.7 → 19.8 | NR | NR | ENMG improved in all | NR → NR | Normal |
| (Sun, Pepijn O. et al., 2020) | 10 | 10 | EN ± HFPF | 0/10 | 0/10 | 8.2 (SD 0.8) → 2.1 (SD 1.4) | Pain improvement 8/10 | NR | NR | NR | SCV/DML improved in 70% (Δ sensory velocity +8 m/s) | 10/10 → 3/10 | Normal retained |
| (Ten Heggeler et al., 2022) | 63 | 63 | EN ± HFPF | 0/63 | 10/63 | NR → 3.0 (SD 0.6) | Pain-free 1.94 (SD 0.73) (reported); ~95% BCTQ improvement | NR | 2.7 (SD 0.7) → 1.92 (SD 0.76) | NR | No standardised post-op EMG | 63/63 → NR | NR |
| (Bollmann et al., 2023) | 36 | 33 | CN ± HFPF | 4/36 | 0/36‡ | NR → NR | Pain improvement 29/36 | NR | NR | NR | SNCV 39.6 → 49.4 m/s; DML 4.0 → 3.5 ms | 31/36 → 5/36 | Normal gliding; no stiffness |
| (Gmainer et al., 2023) | 18 | 18 | EN ± HFPF | 1/18 | 1/18 | 6.7 (SD 2.0) → 2.8 (SD 1.6) | Pain improvement 13/17 | 68 (SD 23) → 24 (SD 19) | NR | NR | DML improvement ~0.7 ms (p < 0.05) | 18/18 → 4/18 | Normal motion achieved |
| Vascularised Flap Coverage: Synovial flap (± skin/Z-plasty adjuncts) |  |  |  |  |  |  |  |  |  |  |  |  |  |
| (Payr et al., 2019) | 15 | 14 | EN ± synovial flap ± Z-plasty/STSG | 0/15 | 0/15 | 8.5 → 2.2 | Pain improvement 11/15 | NR | NR | NR | DML 5.1 → 3.8 ms (−25%, p < 0.05); NCV +2.8 m/s | All positive pre → weakened remnant post | Normal by 2 weeks |
| (Kimura et al., 2025) | 10 | 10 | Synovial flap (SF) | 0/10 | 0/10 | 76.5 (SD 11.1) → 13.0 (SD 10.3) | Pain improvement 10/10 | 52.4 (SD 17.1) → 19.3 (SD 12.7) | NR | NR | DML 8.2 → ~4-6 ms (2 cases); clinical improvement | 9/10 → 0/10 | N/A |
| (Travis et al., 2026) | 17 | 14 | CN + synovial flap (SF) | 3/17 | 0/17 | NR | NR | NR | NR | NR | NR | NR | NR |
| Vascularised Flap Coverage: Other vascularised flaps (non-HFPF) |  |  |  |  |  |  |  |  |  |  |  |  |  |
| (Tham et al., 1996) | 6 | 6 | Reverse-flow RAFFF | 3/6 | 0/6 | NR → NR | Pain improvement 6/6 | NR → NR | NR | NR | DML 5 → 3.5 ms; 2-point discrimination 8 → 4 mm | 5/6 → 0/6 | Normal |
| (Mahmoud et al., 2013) | 8 | 8 | RAFFF | 0/8 | 0/8 | NR → NR | Pain improvement 8/8 | NR | NR | NR | Distal latency 7.5 → 5.5 ms; sensory velocity 25 → 30 m/s | 5/8 → 2/8 | Full ROM |
| (Cheung et al., 2017) | 14 | 12 | EN + pedicled ADMF | 1/14 | 0/14 | 8 → 2.9 (SD 1.5) | Pain improvement 11/14 | NR → 29.4 (SD 26) | NR | NR | NCS improved in all; some normalised | NR → NR | NR |
| (Ratigan et al., 2024) | 5 | 4 | Antebrachial fascia flap + endoscopic TCL release | 0/5 | 0/5 | 6.05 → 3.45 | Pain improvement 5/5 | NR | NR | NR | NCS improved post-revision | 5/5 → 0/5 | Full |
| (Jones et al., 2012) | 18 | 18 | EN ± epineurectomy; HFPF or RFAP | 5/18 | 8/18 | NR → NR | Pain improvement 14/18 | NR | NR | NR | Improved motor conduction and symptoms in ~80% | Positive → resolved in majority (not numeric) | Normal regained |
| Non-Flap Revision: External neurolysis / decompression |  |  |  |  |  |  |  |  |  |  |  |  |  |
| (Duclos and Sokolow, 1998) | 13 | 13 | EN | 0/13 | 0/13 | NR → NR | Pain improvement 12/13 | NR → NR | NR | NR | NCS normalised/improved in 83% (5/6 tested) | 13/13 → 0/13 | Normal ROM; free gliding |
| (Eroğlu et al., 2018) | 23 | 23 | EN + TCL release | 3/23 | 0/23 | NR → NR | Pain improvement 21/23 | NR | NR | NR | Latency improved in all; normalised in 21/23; 2 severe unchanged | 23/23 → 0/23 | Normal |
| (Stirling et al., 2020) | 14 | 13 | EN | 0/14 | 0/14 | NR | Pain improvement 13/14 | 55 (SD 16) → 29 (SD 26) | NR | NR | All had improved NCS post-revision (no numeric) | NR | Full ROM |
| Non-Flap Revision: Neurolysis/decompression with proximal release adjunct |  |  |  |  |  |  |  |  |  |  |  |  |  |
| (Luangjarmekorn et al., 2016) | 25 | 22 | EN ± pronator teres release | 0/25 | 0/25 | NR → NR | Pain improvement 14/25 | NR | NR | NR | NCS/EMG: qualitatively improved | 25/25 → 2/25 | Normal |
|  |  |  |  |  |  |  |  |  |  |  |  |  |  |
| Non-Flap Revision: Autologous vein wrap (AVW) |  |  |  |  |  |  |  |  |  |  |  |  |  |
| (Varitimidis et al., 2000) | 15 | 15 | EN + AVW | 0/15 | 0/15 | 7.5 → 4.0 | Pain improvement 15/15 | NR → NR | NR | NR | Sensory latency 4.5 → 3.0 ms; motor latency 5.5 → 3.8 ms; 2-point discrimination ↓ >2 mm | 15/15 → 1/15 | Maintained |
| (Mulgrew et al., 2012) | 15 | 15 | EN + AVW | 7/15 | 1/15 | 7 → 2 | Pain improvement 15/15 | NR | NR | NR | Motor latency improved ~0.3-0.5 ms; sensory velocity ↑ ~5-10 m/s | Positive in all → resolved in majority | NR |
| Non-Flap Revision: Implant / commercial barrier |  |  |  |  |  |  |  |  |  |  |  |  |  |
| (Bilasy et al., 2012) | 21 | 20 | EN + implant interposition (Canaletto/Eurymed) | 7/21 | 1/21 | 6.45 → 3.86 | Pain improvement 17/21 | 55.3 → 34.96 | NR | NR | NCV 29.8 → 39.1 m/s (+9.3) | 16/21 → 5/21 | N/A |
| Hones (2023) (Hones et al., 2023) | 5 | 5 | CN + VersaWrap hydrogel | 0/5 | 0/5 | 4.2 → NR | 100% subjective/objective improvement | 66.2 → NR | NR | NR | NR | NR | Wrist flex 75°, ext 70°; full finger ROM 3/5 |
| Non-Flap Revision: Tendon interposition |  |  |  |  |  |  |  |  |  |  |  |  |  |
| (Sun, Pepijn Olivier et al., 2020) | 49 | 49 | CN + flexor retinaculum excision + free palmaris longus interposition | 1/49 | 2/49 | 6.2 (SD 1.2) → 2.8 (SD 0.9) | Pain-free 3.8 (SD 0.9) (reported); pain improvement 45/49 | NR | 2.45 (SD 0.6) → 3.5 (SD 0.8) | 2.3 (SD 0.5) → NR | DML improvement reported (~0.9 ms) | 27/49 → 7/49 | NR |
| Non-Flap Revision: Flexor tenosynovectomy / debulking adjunct |  |  |  |  |  |  |  |  |  |  |  |  |  |
| (Kluemper et al., 2021) | 108 | 100 | TCL release + flexor tenosynovectomy | 5/108 | 0/108 | NR → NR | Pain improvement 104/108 | NR → 31.2 | NR | NR | NCS severity improved (non-numeric) | NR → NR | N/A |
| Non-Flap Revision: Autologous fat transfer / grafting |  |  |  |  |  |  |  |  |  |  |  |  |  |
| (Cabrejo et al., 2023) | 81 | 81 | AFT | 2/81 | 0/81 | NR → NR | 92% improved (CTR + fat) vs 50% CTR alone | NR | NR | NR | EMG abnormal in 88% pre (post not quantified) | 81/81 → 0/81 | Full freedom |
| (Gostelie et al., 2026) | 29 | 26 | EN + AFT | 0/29 | 0/29 | NR → NR | Pain-free 3.75 (scale NR); pain improvement 25/29 | NR → 29 | 2.12 → 3.73 | 1.86 → NR | NCS index 0.88 → 0.69; unrecordable → recordable in 5/6 hands | NR | NR |
| Comparative / Mixed Arms |  |  |  |  |  |  |  |  |  |  |  |  |  |
| (Pace et al., 2018) | 23 | 22–23* | Open decompression (OD) ± HFPF (comparative) | 0/23 | 0/23 | NR → NR | Pain improvement 18/23 | NR | Reported as group values only | Reported as group values only | NR | NR | NR |
| (Akrivos et al., 2026) | 14 | 14 | Open decompression (OD) ± HFPF (study arm: OD) | 0/14 | 1/14 | 8 → 1.8 | Pain improvement 12/14 | 65 → 18.5 | NR | NR | 5/5 abnormal pre; 25/30 normalised post (study-level) | 14/14 → 2/14 | N/A |
| (Zieske et al., 2013b) | 19 | 18 | EN ± HFPF/AVW | 1/19 | 1/19 | 5.3 (SD 2.2) → 2.0 (SD 1.7) | Pain improvement 15/19 | NR | NR | NR | DML decreased ~0.8 ms | 12/19 → 4/19 | Strength ↑ (p < 0.01) |

Re-revision values are presented as the number of hands requiring subsequent operative revision after the index revision procedure divided by the number of hands in that study. Crude group-level proportions were calculated from summed events and denominators among studies reporting re-revision data; pooled incidences were estimated separately using random-effects meta-analysis.

Abbreviations: ADMF, abductor digiti minimi flap; AFT, autologous fat transfer; AVW, autologous vein wrap; BCTQ, Boston Carpal Tunnel Questionnaire; CN, complete neurolysis; DML, distal motor latency; EMG/ENMG, electromyography; EN, external neurolysis; HFPF, hypothenar fat pad flap; NCS, nerve conduction studies; NR, not reported; OD, open decompression; RAFFF, radial artery forearm fascial flap; RFAP, radial forearm adipofascial flap; SSS, Symptom Severity Scale; FSS, Functional Status Scale; TCL, transverse carpal ligament.

Table S2. Detailed complication outcomes.

| Author, year | Procedure category | Hands, n | Total complications, n/N hands | Wound complication | Infection | Haematoma / seroma | Scar tenderness / pillar pain | CRPS | Neuroma / nerve injury | Donor-site morbidity | Implant-related complication | Flap compromise / loss | Reoperation for complication |
| --- | --- | --- | --- | --- | --- | --- | --- | --- | --- | --- | --- | --- | --- |
| Vascularised Flap Coverage: HFPF-based ± neurolysis / re-release |  |  |  |  |  |  |  |  |  |  |  |  |  |
| Craft et al., 2007 | HFPF | 28 | 0/28 | 0 | 0 | NR | NR | NR | 0 | NR | N/A | NR | 0 |
| Fusetti et al., 2009 | HFPF | 20 | 2/20 | NR | 1 superficial infection | NR | NR | NR | 1 ulnar border paresthesia | NR | N/A | NR | NR |
| Karthik et al., 2012 | HFPF | 27 | 0/27 | 0 | 0 | NR | NR | NR | NR | NR | N/A | NR | 0 |
| Wichelhaus et al., 2015 | HFPF | 18 | 2/18 | 2 hypertrophic scars | 0 | NR | 2 hypertrophic scars | NR | NR | NR | N/A | NR | 0 |
| Lattré et al., 2016 | HFPF | 8 | 0/8 | 0 | 0 | NR | NR | NR | NR | v | N/A | NR | 0 |
| Athlani and Haloua, 2017 | HFPF | 34 | 0/34 | 0 | 0 | NR | NR | 0 | NR | NR | N/A | NR | 0 |
| Sun, Pepijn O. et al., 2020 | HFPF-based revision procedure | 49 | 1/49 | 1 | 0 | 0 | 0 | 0 | 1 numbness | 0 | 0 | 0 | 1 |
| ten Heggeler et al., 2022 | Revision decompression ± neurolysis ± HFPF | 63 | 2/63 | 1 | 1 | NR | NR | NR | NR | NR | N/A | NR | 0 within 6 mo |
| Bollmann et al., 2023 | Synovial flap | 36 | 4/36 | 1 wound dehiscence | 1 infection | NR | NR | 2 CRPS | NR | N/A | N/A | NR | NR |
| Gmainer et al., 2023 | Revision decompression ± fat flap coverage | 18 | 1/18 | NR | NR | 1 transient seroma | NR | NR | NR | NR | N/A | NR | 0 |
| Vascularised Flap Coverage: Synovial flap ± skin/Z-plasty adjuncts |  |  |  |  |  |  |  |  |  |  |  |  |  |
| Payr et al., 2019 | Synovial flap ± integument enlargement | 15 | 0/15 | 0 skin issue | 0 | NR | NR | NR | NR | N/A | N/A | 0 | 0 |
| Kimura et al., 2025 | Synovial flap | 10 | 0/10 | 0 | 0 | 0 haematoma | NR | NR | NR | N/A | N/A | 0 | 0 |
| Travis et al., 2026 | Synovial flap | 17 | 3/17 | 1 dehiscence; 1 swelling | 1 superficial infection | 1 swelling | NR | NR | NR | N/A | N/A | NR | 0 |
| Vascularised Flap Coverage: Other vascularised flaps, non-HFPF |  |  |  |  |  |  |  |  |  |  |  |  |  |
| Tham et al., 1996 | Other vascularised flap / reverse radial artery fascial flap | 6 | 3/6 | NR | NR | NR | 3 arm scar tenderness | NR | NR | 3 donor-site scar tenderness | N/A | NR | 0 |
| Mahmoud et al., 2013 | Other vascularised flap / radial forearm fascial flap | 8 | 0/8 | 0 | 0 | NR | NR | NR | NR | 0 | N/A | NR | 0 |
| Cheung et al., 2017 | Other vascularised flap / ADMF | 14 | 1/14 | 1 contact dermatitis | NR | NR | NR | NR | NR | Low donor morbidity reported | N/A | NR | No |
| Ratigan et al., 2024 | Endoscopic revision decompression ± antebrachial fascia flap | 5 | 0/5 | 0 | 0 | 0 | NR | NR | NR | NR | N/A | NR | 0 |
| Jones et al., 2012 | Mixed revision decompression ± vascularised coverage | 18 | 5/18 | Minor wound problems not granularly specified | NR | NR | Tenosynovitis / scar-related morbidity reported non-granularly | NR | NR | NR | N/A | NR | 5 |
| Non-Flap Revision: External neurolysis / decompression |  |  |  |  |  |  |  |  |  |  |  |  |  |
| Duclos and Sokolow, 1998 | Repeat decompression / external neurolysis alone | 13 | 0/13 | 0 | 0 | NR | NR | NR | 0 | N/A | N/A | N/A | 0 |
| Eroğlu et al., 2018 | Repeat decompression / revision TCL release | 23 | 3/23 | 2 wound infections | 2 wound infections | 1 haematoma | NR | NR | NR | N/A | N/A | N/A | NR |
| Stirling et al., 2020 | Repeat decompression / neurolysis alone | 14 | 0/14 | 0 | 0 | NR | NR | NR | 0 | N/A | N/A | N/A | 0 |
| Non-Flap Revision: Neurolysis/decompression with proximal release adjunct |  |  |  |  |  |  |  |  |  |  |  |  |  |
| Luangjarmekorn et al., 2016 | Combined wrist and proximal decompression | 25 | 0/25 | 0 | 0 | NR | NR | NR | NR | NR | N/A | N/A | 0 |
| Non-Flap Revision: Autologous vein wrap |  |  |  |  |  |  |  |  |  |  |  |  |  |
| Varitimidis et al., 2000 | Autologous vein wrapping | 15 | 1/15 | NR | NR | NR | NR | NR | NR | 1 donor-leg discomfort lasting up to 1 yr | N/A | N/A | 0 |
| Mulgrew et al., 2012 | Autologous vein wrapping | 15 | 7/15 | NR | NR | NR | 4 long scar concerns | NR | 1 saphenous neuroma | 2 foot swelling; 4 long scar concerns; 1 saphenous neuroma | N/A | N/A | 1 neuroma reoperation |
| Non-Flap Revision: Implant / commercial barrier |  |  |  |  |  |  |  |  |  |  |  |  |  |
| Bilasy et al., 2011 | Synthetic / biologic implant | 21 | 7/21 | Minor suture irritation possible | 0 | NR | 6 pain same or worse | NR | NR | N/A | 1 implant migration requiring removal | N/A | 1 implant removal |
| Hones et al., 2023 | Synthetic / biologic nerve wrap | 5 | 0/5 | 0 | 0 | NR | NR | NR | NR | N/A | 0 | N/A | 0 |
| Non-Flap Revision: Tendon interposition |  |  |  |  |  |  |  |  |  |  |  |  |  |
| Sun, Pepijn Olivier et al., 2020 | Palmaris longus / tendon interposition | 10 | 0/10 | 0 | 0 | NR | NR | NR | NR | 0 | N/A | N/A | 0 |
| Non-Flap Revision: Flexor tenosynovectomy / debulking adjunct |  |  |  |  |  |  |  |  |  |  |  |  |  |
| Kluemper et al., 2019/2021 | Flexor tenosynovectomy | 108 | 5/108 | Wound dehiscence included | Infection included | Haematoma included | NR | NR | NR | N/A | N/A | N/A | 5 |
| Non-Flap Revision: Autologous fat transfer / grafting |  |  |  |  |  |  |  |  |  |  |  |  |  |
| Cabrejo et al., 2023 | Autologous fat grafting adjunct | 81 | 2/81 | NR | 1 cellulitis | NR | NR | NR | NR | 1 abdominal pain after fat harvest | N/A | N/A | 0 |
| Gostelie et al., 2025 | Autologous fat grafting | 29 | 0/29 | 0 | 0 | 0 | NR | NR | NR | No donor-site morbidity reported | N/A | N/A | 0 |
| Comparative / Mixed Arms |  |  |  |  |  |  |  |  |  |  |  |  |  |
| Pace et al., 2018 | Repeat decompression ± HFPF | 23 | 0/23 | 0 | NR | NR | NR | NR | NR | NR | N/A | NR | 0 |
| Akrivos et al., 2025 | HFPF arm | 14 | 0/14 | 0 | 0 | 0 | NR | NR | NR | NR | N/A | NR | 0 |
| Akrivos et al., 2025 | Repeat decompression alone arm | 16 | 0/16 | 0 | 0 | 0 | NR | NR | NR | N/A | N/A | N/A | 0 |
| Zieske et al., 2013 | Mixed revision decompression ± HFPF / vein wrap | 19 | 1/19 | NR | 1 minor infection | NR | NR | NR | NR | NR | N/A | NR | 0 |

Abbreviations: ADMF, abductor digiti minimi flap; CRPS, complex regional pain syndrome; HFPF, hypothenar fat pad flap; N/A, not applicable; NR, not reported; TCL, transverse carpal ligament.

Table S3. Detailed re-revision outcomes.

| Author, year | Initial revision procedure | Hands at risk, n | Re-revisions, n | Time to re-revision | Indication for re-revision | Findings at re-revision | Procedure performed at re-revision | Outcome after re-revision | Follow-up completeness |
| --- | --- | --- | --- | --- | --- | --- | --- | --- | --- |
| Vascularised Flap Coverage: HFPF-based ± neurolysis / re-release |  |  |  |  |  |  |  |  |  |
| Craft et al., 2007 | Microneurolysis + hypothenar fat pad flap | 28 | 0 | N/A | N/A | N/A | N/A | N/A | Mean follow-up 10.5 mo; 28 hands |
| Fusetti et al., 2009 | External neurolysis + hypothenar fat pad flap | 20 | 0 | N/A | N/A | N/A | N/A | N/A | Mean follow-up 13 mo; 20 hands |
| Karthik et al., 2012 | Scar release + hypothenar fat pad flap | 27 | 0 | N/A | N/A | N/A | N/A | N/A | Mean follow-up 22 mo; 27 hands / 25 patients |
| Wichelhaus et al., 2015 | Open revision neurolysis + vascularized hypothenar fat pad flap | 18 | 0 | N/A | N/A | N/A | N/A | N/A | Mean follow-up 22 mo; 18 hands |
| Lattré et al., 2016 | Open revision + pedicled hypothenar fat pad flap | 8 | 0 | N/A | N/A | N/A | N/A | N/A | Follow-up 12 mo; 8 hands |
| Athlani and Haloua, 2017 | Revision neurolysis + pedicled hypothenar fat pad flap | 34 | 0 | N/A | N/A | N/A | N/A | N/A | Minimum follow-up 24 mo; 13 patients followed to 5 yrs |
| Sun, Pepijn O. et al., 2020 | HFPF-based revision procedure | 49 | 0 | 0 | 0 | 0 | 0 | 0 | Follow-up 6 mo; 49 hands |
| Ten Heggeler et al., 2022 | Open revision CTR with external / internal neurolysis ± fat pad flap | 63 | 0 within follow-up | N/A | N/A | N/A | N/A | N/A | Follow-up questionnaire at 6 mo; 63 revision hands |
| Bollmann et al., 2023 | Open revision neurolysis + vascularized synovial flap | 36 | 0 | N/A | N/A | N/A | N/A | N/A | Mean follow-up 11.4 mo; 36 hands / 33 patients |
| Gmainer et al., 2023 | External neurolysis ± fat flap coverage | 18 | 1 | NR | Persistent or recurrent symptoms after revision decompression | NR | NR | NR | Mean follow-up 18 ± 8 mo; 18 recurrent CTS hands |
| Vascularised Flap Coverage: Synovial flap ± skin/Z-plasty adjuncts |  |  |  |  |  |  |  |  |  |
| Payr et al., 2019 | Neurolysis + Wulle synovial flap ± integument enlargement | 15 | 0 | N/A | N/A | N/A | N/A | N/A | Follow-up 12 mo; 15 hands / 14 patients |
| Kimura et al., 2025 | Carpal tunnel re-release + vascularised flexor synovial flap wrap | 10 | 0 | N/A | N/A | N/A | N/A | N/A | Mean follow-up 44.8 mo; 10 hands |
| Travis et al., 2026 | Revision open CTR + vascularized synovial flap | 17 | 0 | N/A | N/A | N/A | N/A | N/A | Mean follow-up approximately 40 mo; 17 hands / 14 patients |
| Vascularised Flap Coverage: Other vascularised flaps, non-HFPF |  |  |  |  |  |  |  |  |  |
| Tham et al., 1996 | Reverse-flow radial artery forearm fascial flap | 6 | 0 | N/A | N/A | N/A | N/A | N/A | Mean follow-up 25 mo; 6 hands |
| Mahmoud et al., 2013 | Neurolysis + perforator-based radial forearm fascial flap | 8 | 0 | N/A | N/A | N/A | N/A | N/A | Mean follow-up 20 mo; 8 hands |
| Cheung et al., 2017 | External neurolysis + pedicled abductor digiti minimi flap | 14 | 0 | N/A | N/A | N/A | N/A | N/A | Mean follow-up 44 mo; 14 hands / 12 patients |
| Ratigan et al., 2024 | Endoscopic revision CTR | 5 | 0 | N/A | N/A | N/A | N/A | N/A | Follow-up 6 wk; 5 hands / 4 patients |
| Jones et al., 2012 | Extended revision decompression + external neurolysis ± epineurectomy ± flap coverage | 18 recurrent hands | 5 patients in overall series required third operation; recurrent-specific n unclear | NR | Persistent symptoms / failed revision surgery | NR | Third operation; exact procedure NR | NR | Minimum follow-up 12 mo; recurrent subgroup 18 hands |
| Non-Flap Revision: External neurolysis / decompression |  |  |  |  |  |  |  |  |  |
| Duclos and Sokolow, 1998 | Extensive external neurolysis only | 13 | 0 | N/A | N/A | N/A | N/A | N/A | Mean follow-up 27.5 mo; 13 hands |
| Eroğlu et al., 2018 | Mini-open revision CTR with complete TCL release | 23 | 0 | N/A | N/A | N/A | N/A | N/A | Mean follow-up 8.6 mo; 23 hands |
| Stirling et al., 2020 | Open revision decompression / neurolysis only | 14 | 0 | N/A | N/A | N/A | N/A | N/A | Mean follow-up 20 mo; 14 hands / 13 patients |
| Non-Flap Revision: Neurolysis/decompression with proximal release adjunct |  |  |  |  |  |  |  |  |  |
| Luangjarmekorn et al., 2016 | Revision CTR alone or revision CTR + pronator teres release | 25 | 0 | N/A | N/A | N/A | N/A | N/A | Follow-up at least 6 mo; 25 hands / 22 patients |
| Non-Flap Revision: Autologous vein wrap |  |  |  |  |  |  |  |  |  |
| Varitimidis et al., 2000 | External neurolysis + autologous saphenous vein wrap | 15 | 0 | N/A | N/A | N/A | N/A | N/A | Mean follow-up 43 mo; 15 hands |
| Mulgrew et al., 2012 | External neurolysis + autologous great saphenous vein wrap | 15 | 1 donor-site reoperation | NR | Symptomatic donor-site saphenous neuroma | Saphenous nerve neuroma at donor site | Reoperation for neuroma; burial of nerve end described as necessary | NR | Mean follow-up 37 mo; 15 upper-limb neuropathy cases |
| Non-Flap Revision: Implant / commercial barrier |  |  |  |  |  |  |  |  |  |
| Bilasy et al., 2011/2012 | External neurolysis + Canaletto implant interposition | 21 | 1 | NR | Implant migration with persistent lack of clinical benefit | Implant migration | Implant removal | No benefit after removal | Mean follow-up 12.1 mo; 21 hands / 20 patients |
| Hones et al., 2023 | Re-exploration + neurolysis + VersaWrap nerve protector | 5 | 0 | N/A | N/A | N/A | N/A | N/A | Mean follow-up 4.6 mo; 5 recurrent CTS hands |
| Non-Flap Revision: Tendon interposition |  |  |  |  |  |  |  |  |  |
| Sun, Pepijn Olivier et al., 2020 | Neurolysis + free palmaris longus tendon interposition graft | 10 | 0 | N/A | N/A | N/A | N/A | N/A | Follow-up 12 mo; 10 recurrent CTS hands |
| Non-Flap Revision: Flexor tenosynovectomy / debulking adjunct |  |  |  |  |  |  |  |  |  |
| Kluemper et al., 2019/2021 | Open revision CTR + flexor tenosynovectomy | 108 | 0 for recurrent CTS; 5 reoperations for complications | Early postoperative period; exact timing NR | Wound dehiscence, infection, or haematoma requiring return to operating room | NR | Return to operating room for wound / infection / haematoma management | NR | Clinical follow-up 12 mo plus survey mean 81 mo |
| Non-Flap Revision: Autologous fat transfer / grafting |  |  |  |  |  |  |  |  |  |
| Cabrejo et al., 2023 | Revision CTR alone or revision CTR + autologous fat grafting | 81 | 0 | N/A | N/A | N/A | N/A | N/A | Mean follow-up approximately 17 mo; 81 hands |
| Gostelie et al., 2025/2026 | Extensive external neurolysis + autologous perineural fat grafting | 29 | 0 | N/A | N/A | N/A | N/A | N/A | Median follow-up 5.3 yrs; 29 hands / 26 patients |
| Comparative / Mixed Arms |  |  |  |  |  |  |  |  |  |
| Pace et al., 2018 | Repeat decompression alone or repeat decompression + hypothenar fat pad transposition | 23 | 0 | N/A | N/A | N/A | N/A | N/A | Mean survey follow-up approximately 53 mo; 23 recurrent CTS hands |
| Akrivos et al., 2025/2026 | Group A: open revision + hypothenar fat pad flap; Group B: re-open decompression only | 30 total; 14 HFPF and 16 decompression-only in supplied data | 1 total; 0 HFPF, 1 decompression-only | NR | Recurrent symptoms / recurrence after revision decompression | NR | NR | NR | Long-term follow-up approximately 132–144 mo |
| Zieske et al., 2013 | External neurolysis ± hypothenar fat pad flap / vein wrap | 19 recurrent hands | 1 | NR | Persistent or recurrent symptoms after revision surgery | NR | NR | NR | Mean follow-up 14 ± 9 mo; 19 recurrent CTS hands |

Abbreviations: CTR, carpal tunnel release; CTS, carpal tunnel syndrome; HFPF, hypothenar fat pad flap; mo, months; N/A, not applicable; NR, not reported; TCL, transverse carpal ligament; wk, weeks; yrs, years.

Re-revision was defined as any additional operative intervention after the index revision procedure when reported. Where studies described reoperation for wound complications, implant migration, or donor-site morbidity, these were retained in the table for transparency but should not be pooled as recurrent CTS durability failures unless the study explicitly attributed the reoperation to recurrent median nerve compression.

Table S4. Diagnostic work-up and proximal compression.

| Author, year | Recurrent vs persistent distinguished | Symptom-free interval reported | NCS/EMG performed preoperatively | Ultrasound performed | MRI performed | Cervical radiculopathy assessed | Pronator/proximal median compression assessed | Proximal compression identified, n/N | Proximal decompression performed, n | Outcome separately reported for proximal compression subgroup | Notes |
| --- | --- | --- | --- | --- | --- | --- | --- | --- | --- | --- | --- |
| Vascularised Flap Coverage: HFPF-based ± neurolysis / re-release |  |  |  |  |  |  |  |  |  |  |  |
| Craft et al., 2007 | Yes | Yes; true recurrent CTS after symptom-free period of several months to years | Yes | NR | NR | NR | NR | 0/28 reported | 0 | No | Polyneuropathy reported as absent in 0/28; proximal compression not otherwise assessed or reported. |
| Fusetti et al., 2009 | Yes | Yes; mean 17 months since previous surgery | Yes; ENMG reported, with postoperative improvement in a subset | NR | NR | NR | NR | NR | NR | No | Study focused on recurrent CTS treated with external neurolysis and HFPF; workers’ compensation reported in 9/20, but proximal compression work-up not described. |
| Karthik et al., 2012 | Yes | Yes; true recurrence after >3 months relief, mean interval approximately 56 months | Yes | NR | NR | Mentioned as prognostic factor, but not systematically quantified | NR | NR | NR | No | Cervical spine problems were mentioned as associated with poorer or delayed recovery, but no denominator or formal diagnostic pathway was reported. |
| Wichelhaus et al., 2015 | Yes | Yes; recurrent CTS 5-22 months after prior release | Yes | NR | NR | NR | NR | NR | NR | No | Nerve conduction velocity and latency were reported, but proximal compression was not described. |
| Lattré et al., 2016 | Yes | Yes; recurrent CTS ≥1 year after primary open release | Yes; EMG confirmed compression preoperatively | NR | NR | NR | NR | NR | NR | No | Focused on recurrent/end-stage CTS treated with HFPF; no proximal compression assessment reported. |
| Athlani and Haloua, 2017 | Mixed recurrent CTS and/or neuropathic pain after failed CTR | Yes; mean 61 months from prior procedure | Yes; abnormal ENMG required | NR | NR | NR | NR | NR | NR | No | Required abnormal ENMG but did not report systematic cervical or proximal median nerve evaluation. |
| Sun, Pepijn O. et al., 2020 | Yes | Yes; recurrent CTS defined by symptom-free interval before recurrence | Yes | NR | NR | NR | NR | 4/20 prior cubital tunnel syndrome | NR | No | Dataset reports prior cubital tunnel syndrome in 4/20; whether this was actively assessed as part of the revision work-up was not clear. |
| ten Heggeler et al., 2022 | Yes; recurrent and persistent subgroups distinguished | Yes; recurrent CTS defined as ≥6 months symptom-free after primary release | No standardized postoperative EMG; preoperative electrodiagnostic details not granularly reported | NR | NR | NR | NR | 0/98 hand/wrist/proximal compression reported in comorbidity table | 0 | No | Registry study included recurrent and persistent revision cases; comorbidities were grouped, and proximal compression was not a major analytic focus. |
| Bollmann et al., 2023 | Yes | Yes; true recurrent CTS after symptom-free period ≥3 months | Yes | NR | NR | 1/36 cervical/double-crush reported | Other nerve compression assessed or treated in selected cases | 9/36 with other proximal/concomitant nerve compression procedures | 9 | No | Other proximal/concomitant nerve procedures included ulnar nerve procedures and radial nerve neurolysis; outcomes were not separately reported for this subgroup. |
| Gmainer et al., 2023 | Yes; recurrent and persistent pain groups compared | Yes; mean interval approximately 25 months | Yes | NR | NR | 0/18 cervical/double-crush reported | NR | 0/18 reported | 0 | No | Cervical/double-crush reported as absent in the extracted comorbidity data; no dedicated proximal compression subgroup outcome reported. |
| Vascularised Flap Coverage: Synovial flap ± skin/Z-plasty adjuncts |  |  |  |  |  |  |  |  |  |  |  |
| Payr et al., 2019 | Yes | Yes; true recurrence after symptom-free period >3 months | Yes | NR | NR | 0/14 reported | NR | 0/14 reported | 0 | No | No cervical radiculopathy or polyneuropathy reported in the extracted data. |
| Kimura et al., 2025 | Yes | Yes; recurrent CTS after ≥1 prior CTR, mean 11.9 months | Yes | NR | NR | NR | NR | NR | NR | No | No diabetes, rheumatoid arthritis, or renal dialysis/amyloid reported; proximal compression not described. |
| Travis et al., 2026 | Yes | Yes; recurrent CTS after ≥12 months symptom-free | Not recorded / NR | NR | NR | 1/14 cervical/double-crush reported | Cubital tunnel syndrome reported | 2/14 cubital tunnel syndrome; 1/14 cervical/double-crush | NR | No | Cubital tunnel syndrome was reported in 2/14 and cervical/double-crush in 1/14, but subgroup-specific outcomes were not reported. |
| Vascularised Flap Coverage: Other vascularised flaps, non-HFPF |  |  |  |  |  |  |  |  |  |  |  |
| Tham et al., 1996 | Yes | Yes; recurrent CTS after ≥2 prior CTRs | Yes; EMG reported | NR | NR | NR | NR | NR | NR | No | Workers’ compensation reported in 2/6; proximal compression work-up not described. |
| Mahmoud et al., 2013 | Yes | Yes; recurrent CTS after ≥1 prior CTR | Yes | NR | NR | NR | NR | NR | NR | No | Focused on radial forearm fascial flap coverage; no proximal compression assessment reported. |
| Cheung et al., 2017 | Yes | NR | Yes; NCS improvement reported | NR | NR | 1/12 ulnar brachial plexopathy reported; cervical/double-crush otherwise not reported | NR | 1/12 ulnar brachial plexopathy | NR | No | Complex recurrent CTS/CRPS cohort; one patient had ulnar brachial plexopathy. Outcomes were not separately reported for this patient. |
| Ratigan et al., 2024 | Yes | Yes; recurrent CTS 2–30 years after open CTR | Yes | NR | NR | NR | NR | NR | NR | No | At least 2/5 cases had diffuse peripheral polyneuropathy on EMG; one additional case may also have had severe diffuse polyneuropathy depending on interpretation. |
| Jones et al., 2012 | Yes; persistent and recurrent CTS distinguished | Yes; recurrence after initial symptom relief, mean interval approximately 21 months | Yes | NR | NR | Yes; cervical radiculopathy reported | Yes; pronator syndrome and other proximal compression reported | 10/18 with cubital/ulnar tunnel/pronator syndrome; 4/18 cervical/double-crush | At least 10 reported proximal nerve compression diagnoses/procedures in recurrent subset | No | Proximal/concomitant diagnoses included cubital tunnel syndrome, ulnar tunnel syndrome, pronator syndrome, and cervical radiculopathy; subgroup-specific outcomes were not reported. |
| Non-Flap Revision: External neurolysis / decompression |  |  |  |  |  |  |  |  |  |  |  |
| Duclos and Sokolow, 1998 | Yes | Yes; true recurrent CTS after complete prior release and scarring | Yes | NR | NR | NR | NR | NR | NR | No | Rheumatoid arthritis and workers’ compensation were absent in this small series; proximal compression not reported. |
| Eroğlu et al., 2018 | Yes | Yes; mean 38 weeks from last CTR | Yes; EMG performed | NR | NR | NR | NR | NR | NR | No | Polyneuropathy reported in 10/23, but no cervical or proximal median nerve compression assessment was reported. |
| Stirling et al., 2020 | Yes | Yes; recurrent CTS after >3 months symptom-free and NCS confirmation | Yes; NCS confirmation required | NR | NR | NR | NR | NR | NR | No | Workers’ compensation absent in 0/13; proximal compression not described. |
| Non-Flap Revision: Neurolysis/decompression with proximal release adjunct |  |  |  |  |  |  |  |  |  |  |  |
| Luangjarmekorn et al., 2016 | Failed CTR cohort with suspected pronator teres syndrome; recurrent/persistent distinction not clearly separated in extracted data | NR / mean approximately 50 months from primary procedure | Qualitatively reported / NR granularly | NR | NR | Yes; 2/25 cervical spine problems reported | Yes; proximal median/pronator compression assessed by design | 25/25 suspected pronator teres syndrome; 2/25 cervical spine problems | PTR performed in proximal-release group; exact n NR in supplied dataset | Yes | This was the only focused study of proximal median nerve release; revision CTR plus pronator teres release had higher full recovery of pain and numbness than revision CTR alone. |
| Non-Flap Revision: Autologous vein wrap |  |  |  |  |  |  |  |  |  |  |  |
| Varitimidis et al., 2000 | Yes | NR; recurrent CTS after ≥2 decompressions | Yes | NR | NR | NR | NR | NR | NR | No | Recurrent compressive neuropathy treated with saphenous vein wrapping; proximal compression work-up not reported. |
| Mulgrew et al., 2012 | Mixed recurrent upper-limb neuropathies; recurrent CTS-specific distinction not isolated | NR | Yes; NCV data reported | NR | NR | NR | NR | NR | NR | No | Heterogeneous median, ulnar, and radial nerve neuropathy cohort; proximal compression subgroup outcomes for CTS were not separately reported. |
| Non-Flap Revision: Implant / commercial barrier |  |  |  |  |  |  |  |  |  |  |  |
| Bilasy et al., 2011/2012 | Yes | Yes; mean symptom-free interval approximately 112 weeks | Yes | NR | NR | NR | NR | NR | NR | No | Polyneuropathy reported in 4/20; proximal compression not reported. |
| Hones et al., 2023 | Mixed recurrent compressive neuropathies; recurrent CTS subset included | NR | Not performed for CTS subset / NR | NR | NR | NR | Other proximal compression present by design in overall cohort | 15/20 overall had non-carpal compressive neuropathy treated; CTS subset 5/20 | 15/20 overall non-carpal decompressions | No CTS-specific proximal subgroup outcome | Overall VersaWrap cohort included revision cubital tunnel and radial tunnel cases; recurrent CTS cases were not analyzed separately for proximal compression. |
| Non-Flap Revision: Tendon interposition |  |  |  |  |  |  |  |  |  |  |  |
| Sun, Pepijn Olivier et al., 2020 | Yes; recurrent and persistent CTS included | Yes; recurrent CTS defined by symptom-free interval followed by return of pain/paresthesia | Yes | NR | NR | 0/20 reported | Prior cubital tunnel syndrome reported | 4/20 prior cubital tunnel syndrome | NR | No | Palmaris longus interposition cohort reported prior cubital tunnel syndrome in 4/20 and no cervical/double-crush in 0/20. |
| Non-Flap Revision: Flexor tenosynovectomy / debulking adjunct |  |  |  |  |  |  |  |  |  |  |  |
| Kluemper et al., 2019/2021 | Yes | Yes; recurrent idiopathic CTS after symptom-free period >1 year | Yes; NCS severity reported | NR | NR | NR | Other proximal compression reported as absent | 0/108 prior cubital tunnel / other proximal compression | 0 | No | Large recurrent CTS tenosynovectomy cohort; no proximal compression reported in the extracted comorbidity data. |
| Non-Flap Revision: Autologous fat transfer / grafting |  |  |  |  |  |  |  |  |  |  |  |
| Cabrejo et al., 2023 | Yes | NR / mean interval approximately 75 months from primary procedure | Yes; EMG abnormal in 88% preoperatively | NR | NR | NR | NR | NR | NR | No | Study compared re-CTR with versus without fat grafting; proximal compression assessment not reported. |
| Gostelie et al., 2025/2026 | Recalcitrant CTS after ≥2 prior CTRs; recurrent/persistent distinction not clearly separated in extracted data | NR | Yes; NCS reported | NR | NR | 0/29 reported | 0/29 prior cubital/proximal compression reported | 0/29 | 0 | No | Long-term autologous fat grafting series; cervical/double-crush and other proximal compression were absent in extracted comorbidity data. |
| Comparative / Mixed Arms |  |  |  |  |  |  |  |  |  |  |  |
| Pace et al., 2018 | Yes; recurrent and persistent groups compared | Yes; recurrent CTS defined as return of symptoms after initial relief; mean interval 74 months | No; EMG data not collected | NR | NR | NR | NR | NR | NR | No | Telephone BCTQ study; no preoperative electrodiagnostic or proximal compression work-up details were available in extracted data. |
| Akrivos et al., 2025/2026 | Yes | Yes; recurrent CTS after ≥3 months symptom-free, mean approximately 41 months | Yes; NCS performed | NR | NR | NR | NR | NR | NR | No | Compared HFPF with repeat decompression alone; proximal compression not described. |
| Zieske et al., 2013 | Yes; revision subgroups reported | Yes; recurrent CTS after previous release, interval approximately 30 months in recurrent subgroup | Yes | NR | NR | Yes; 5/19 cervical/double-crush reported in recurrent subset | Yes; cubital tunnel decompression reported | 5/36 cubital tunnel decompressions in overall revision cohort; 5/19 cervical/double-crush in recurrent subset | 5 cubital tunnel decompressionss in overall cohort | No | Proximal compression was recorded, but outcomes were not separately reported for patients with cervical disease or concomitant cubital tunnel decompression. |

Abbreviations: CTS, carpal tunnel syndrome; CTR, carpal tunnel release; EMG, electromyography; HFPF, hypothenar fat pad flap; MPS, mucopolysaccharidosis; MRI, magnetic resonance imaging; NCS, nerve conduction studies; NR, not reported; PTR, pronator teres release.

Table S5. CTS-specific sensory symptoms reported after revision carpal tunnel surgery.

| Study ID | Residual numbness, n | Residual paresthesia, n | Residual nocturnal awakening, n |
| --- | --- | --- | --- |
| Fusetti_2009 | 0 | 3 | 3 |
| Cheung_2017 | 0 | 0 | 0 |
| Kimura_2025 | 0 | 0 | 0 |
| Akrivos_2025a | 0 | 0 | 0 |
| Craft_2007 | 16 | 0 | 0 |
| Mahmoud_2013 | 2 | 0 | 2 |
| Bollmann_2023 | 0 | 0 | 0 |
| Karthik_2012 | 4 | 0 | 0 |
| Tham_1996 | 1 | 4 | 0 |
| Athlani_2017 | 13 | 3 | 3 |
| Payr_2019 | 0 | 0 | 0 |
| Travis_2026 | 0 | 0 | 0 |
| Wichelhaus_2015 | 0 | 0 | 0 |
| Lattre_2016 | 0 | 0 | 0 |
| Gostelie_2025 | 0 | 0 | 0 |
| Krzesniak_2015 | 2 | 2 | 0 |
| Bilasy_2011 | 5 | 0 | 0 |
| Stirling_2020 | 0 | 0 | 0 |
| Kluemper_2019 | 0 | 0 | 0 |
| Mulgrew_2012 | 0 | 0 | 0 |
| Ten_2022 | 0 | 0 | 0 |
| Duclos_1998 | 0 | 2 | 4 |
| Hones_2023 | 0 | 0 | 0 |
| Sun_2020a | 0 | 0 | 0 |
| Gmainer_2023 | 2 | 3 | 2 |
| Sun_2020b | 0 | 0 | 0 |
| Eroglu_2018 | 0 | 0 | 0 |
| Varitimidis_2000 | 0 | 0 | 0 |
| Zieske_2013 | 4 | 0 | 0 |
| Ratigan_2024 | 0 | 0 | 0 |
| Jones_2012 | 10 | 10 | 0 |
| Luangjarmekorn_2016 | 2 | 0 | 0 |
| Pace_2018 | 0 | 0 | 0 |
| Hulsizer_1998 | 0 | 0 | 0 |
| Cabrejo_2023 | 0 | 0 | 0 |
| Akrivos_2025b | 0 | 0 | 0 |
| Total | 63 | 27 | 21 |

Table S6. ROBINS-I assessment of studies.

| Study (Author, Year) | Bias due to Confounding | Bias in Selection of Participants | Bias in Classifications of Interventions | Bias due to Deviations from Intended Interventions | Bias due to Missing Data | Bias in Outcome Measurements | Bias in Selection of Reported Results | Overall Judgement |
| --- | --- | --- | --- | --- | --- | --- | --- | --- |
| Akrivos, 2026 | Moderate Risk; observational study + group allocation is not randomised | Moderate Risk; no random allocation + possible clinician-driven selection could favour one surgical procedure | Low Risk | Low Risk | Low Risk | Moderate Risk; no blinding of assessors | Low Risk | Moderate Risk |
| Lattré, 2016 | Serious Risk; patients were assgined to groups based on clinical status + no randomisation + no formal adjustment for confounders | Moderate Risk | Low Risk | Low Risk | Moderate Risk; does not mention drop-outs/missing data | Moderate Risk; blinding of assessors was not mentioned | Low Risk | Serious Risk |
| ten Heggeler, 2022 | Moderate Risk; residual confounding from unmeasured variables (e.g. surgeon experience, exact surgical technique, psychosocial factors) | Low Risk | Low Risk | Low Risk | Moderate Risk; does not fully specify handling of missing data | Moderate Risk; no mention of assessor blinding | Low Risk | Moderate Risk |
| Gmainer, 2023 | Serious Risk; no randomisation + no strong adjustment for key prognostic variables | Moderate Risk; ronly patients who proceeded to revision srgery were included | Moderate Risk; retrospective symptom classification | Low Risk | Moderate Risk; retrospective data collection is likely to result in missing data | Moderate Risk; use of some subjective outcome measures + no assessor blinding | Moderate Risk; no selection protocol detailed | Serious Risk |
| Sun, 2020 | Moderate Risk; no randomisation + likely unidentified confounding variables | Moderate Risk; only patietns undergoing revision surgery included | Moderate Risk; retrospective symptom classification | Low Risk | Moderate Risk; retrospective + follow up at 6 months means likely missing data | Moderate Risk; subjective outcome measures + no blinding | Moderate Risk; no pre-registered selection protocol reported | Moderate Risk |
| Varitimidis, 2000 | Moderate Risk; not all potential confounders were controlled | Moderate Risk; only patients who underwent revision surgery were included | Low Risk | Low Risk | Moderate Risk; follow up for some patients were incomplete | Moderate Risk; subjective outcome measures + no blinding | Moderate Risk; no pre-registered selection protocol reported | Moderate Risk |
| Wellborn, 2025 | Moderate Risk; residual confounding remains possible | Moderate Risk; retrospective inclusion of only patients who underwent revision endoscopic surgery | Low Risk | Low Risk | Moderate Risk; some incomplete follow ups could have resulted in missing data | Moderate Risk; some subjective outcome measures + no blinding | Moderate Risk; no pre-registered selection protocol | Moderate Risk |
| Zieske, 2013 | Moderate Risk; use of multivariable regression but likely residual confounding | Moderate Risk; retrospective inclusion of only patients who underwent revision surgery | Low Risk | Low Risk | Moderate Risk; some incomplete follow ups could have resulted in missing data | Moderate Risk; some subjective outcome measures + no blinding | Moderate Risk; no pre-registered selection protocol | Moderate Risk |
| Luangjarmekorn, 2016 | Serious Risk; groups formed retrospectively without randomisation + no adjustment for prognostic factors | Moderate Risk; selected based on clinical suspicion of pronator syndrome/possible clinician selection + no randomisation | Low Risk | Low Risk | Moderate Risk; no mention of missing data + some outcomes are inconsistently reported | Moderate Risk; some subjective outcome measures + no blinding | Moderate Risk; only certain outcomes were reported + no pre-registered selection protocol | Serious Risk |
| Pace, 2018 | Serious Risk; no randomisation/based on clinician judgement + no adjustment for confounders | Moderate Risk; retrospective data + single institution + selection based on response availability + does not clearly state "consecutive patients" | Low Risk | Low Risk | Moderate Risk; not all eligible patients completed questionnaires + no handling of missing data described | Moderate Risk; some subjective outcome measures + no blinding | Moderate Risk; no pre-registered selection protocol + unclear if all outcomes were reported | Serious Risk |
| Hulsizer, 1998 | Serious Risk; groups based on prior intervention + no adjustment for confounders | Moderate Risk; selected based on prior intervention + inclusion criteria/recruitment methods not fully described | Low Risk | Low Risk | Moderate Risk; completion of follow up is unclear + missing data is not well reported | Serious Risk; outcomes were self-reported and therefore subjective and unblinded | Moderate Risk; selective reporting of favourable outcomes cannot be ruled out | Serious Risk |
| Cabrejo, 2023 | Serious Risk; groups determined by intervention with no randomisation + limited adjustment for baseline differences | Moderate Risk; retrospective inclusion + likely influenced by clinical judgement | Low Risk | Low Risk | Moderate Risk; completion of follow up not fully reported | Serious Risk; outcomes were patient-reported and likely unblinded | Moderate Risk; selective reporting cannot be ruled out | Serious Risk |
| Lane, 2020 | Serious Risk; groups determined by intervention with no randomisation + limited adjustment for baseline differences | Moderate Risk; retrospective inclusion + likely influenced by clinical judgement | Low Risk | Low Risk | Moderate Risk; completion of follow up not fully reported | Serious Risk; outcomes were patient-reported and likely unblinded | Moderate Risk; selective reporting cannot be ruled out | Serious Risk. |

Table S7. NIH quality assessment of studies.

| Study (Author, Year) | Was the study question or objective clearly stated? | Was the study population clearly and fully described, including a case definition? | Were the cases consecutive? | Were the subjects comparable? | Was the intervention clearly described? | Were the outcome measures clearly defined, valid, reliable, and implemented consistently across all participants? | Was the length of follow-up adequate? | Were the statistical methods well-described? | Were the results well-described? | Overall Scoring (/9) |
| --- | --- | --- | --- | --- | --- | --- | --- | --- | --- | --- |
| Fusetti, 2009 | Yes | Yes | Yes | NA; no control group | Yes | CD; paper does not clearly describe standardised protocols for how each measure is assessed | Yes | No; limited description | Yes | 6 |
| Cheung, 2017 | Yes | Yes | Yes | NA; no control group | Yes | CD; specific measurement methods, assessment timing and blinding not folly described | Yes | No; only descriptive outcomes mentioned | Yes | 6 |
| Gostelie, 2025 | Yes | Yes | Yes | NA; no control group | Yes | Yes | Yes | CD; reports changes in median SSS and FSS scores but does not fully describe statistical testing/modelling | Yes | 7 |
| Krześniak, 2015 | Yes | Yes | Yes | NA; no control group | Yes | CD; outcomes measured using Boston Carpal Tunnel Questionnaire but does not fully detail standardisation of measurement procedures (e.g. blinding) | Yes | No; does not describe handling of missing data, any other statistical testing besides Student's t-test, checking for assumptions | Yes | 6 |
| Bilasy, 2011 | Yes | Yes | Yes | NA; no control group | Yes | CD; outcome measures are not all fully validated, assessment methods are not fully standardised/blinded | Yes | No; primarily descriptive statistics reported, no description of handling missing data or assumptions | Yes | 6 |
| Kimura, 2024 | Yes | Yes | NR | NA; no control group | Yes | CD; standardisation and blinding procedures not fully described | Yes | CD; no detailed description of handing assumptions or missing data | Yes | 5 |
| Stirling, 2019 | Yes | Yes | CD; does not explicitly state "consecutive patients" | NA; no control group | Yes | CD; standardisation and blinding procedures are not fully described | Yes | CD; descriptive statistics reported but no detailed explanation of how assumptions, missing data and variance handling | Yes | 5 |
| Kluemper, 2019 | Yes | Yes | Yes | NA; no control group | Yes | Yes | Yes | Yes | Yes | 8 |
| Mulgrew, 2012 | Yes | Yes | Yes | NA; no control group | Yes | Yes | Yes | Yes | Yes | 8 |
| Craft, 2007 | Yes | Yes | Yes | NA; no control group | Yes | CD; mostly subjective patient-reported improvements + outcomes can vary by how the examiner elicited them | Yes | No; no formal statistical analyses | Yes | 6 |
| Duclos, 1998 | Yes | Yes | Yes | NA; no control group | Yes | CD; outcomes reported were subjective and not standardised | Yes | No; no formal statistical analyses | Yes | 6 |
| Hones, 2023 | Yes | Yes | Yes | NA; no control group | Yes | CD; outcomes were mostly subjective and patient-reported with no standardisation/use of validated instruments | Yes | No; descriptive outcomes only | Yes | 6 |
| Sun, 2019 | Yes | Yes | Yes | NA; no control group | Yes | CD; mostly subjective with no use of standardised validated scoring system | Yes | No; descriptive outcomes only | Yes | 6 |
| Mahmoud, 2013 | Yes | Yes | Yes | NA; no control group | Yes | CD; mostly subjective outcomes with no use of validated instruments or scoring system | Yes | No; descriptive outcomes only | Yes | 6 |
| Bollmann, 2023 | Yes | Yes | No; does not specify that consecutive eligible patients were included | NA; no control group | Yes | CD; subjective outcomes + no assessor blinding | Yes | No; descriptive outcomes only | Yes | 5 |
| Tham, 1995 | Yes | Yes | No; does not specify that consecutive eligible patients were included | NA; no control group | Yes | CD; outcomes are categorical/descriptive + no validated scoring systems use | Yes | No; descriptive outcomes only + no validated scoring systems used | Yes | 5 |
| Jones, 2012 | Yes | Yes | Yes | NA; no control group | Yes | CD; did not use validated outcome measurement instruments + no standardisation/blinding described | NR | No; descriptive outcomes only + no description of statistical analysis | Yes | 5 |
| Athlani, 2017 | Yes | Yes | Yes | NA; no control group | Yes | CD; did not use validated outcome measurement instruments + no standardisation/measurement procedure described | Yes | No; descriptive outcomes only + no description of handling missing data + no formal statistical analysis | Yes | 6 |
| Payr, 2019 | Yes | Yes | NR | NA; no control group | Yes | CD; no mention of blinding | Yes | Yes | Yes | 6 |
| Travis, 2026 | Yes | Yes | NR | NA; no control group | Yes | CD; subjective outcomes + no blinding | Yes | CD; descriptive statistics + unclear if more detaild statistical tests were applied | Yes | 5 |
| Wichelhaus, 2015 | Yes | Yes | Yes | NA; no control group | Yes | CD; standardised tools were used + no blinding | Yes | Yes | Yes | 7 |

Table S8. JBI critical appraisal of studies.

| Study (Author, Year) | Were there clear criteria for inclusion in the case series? | Was the condition measured in a standard, reliable way for all participants included in the case series? | Were valid methods used for identification of the condition for all participants included in the case series? | Did the case series have consecutive inclusion of participants? | Did the case series have complete inclusion of participants? | Was there clear reporting of the demographics of the participants in the study? | Was there clear reporting of clinical information of the participants? | Were the outcomes or follow‑up results of cases clearly reported? | Was there clear reporting of the presenting site(s)/clinic(s) demographic information? | Was statistical analysis appropriate? | Overall Scoring (/10) |
| --- | --- | --- | --- | --- | --- | --- | --- | --- | --- | --- | --- |
| Eroğlu, 2018 | Yes | Yes | Yes | No; does not specify that all recurrent CTS cases were consecutively included + selection appears partial | No; only a subset of recurrent CTS patients were analysed | Yes | Yes | Yes | Yes | NA; no statistical analysis was performed | 7 |
| Karthik, 2011 | Yes | Yes | Yes | No; does not specify that all eligible cases were included consecutively | No; not all recurrent CTS patients were accounted for + no full description of selection process | Yes | Yes | Yes | Yes | NA; no statistical analysis was performed | 7 |

Search strategy

(("carpal tunnel syndrome"[MeSH Terms] OR "carpal tunnel"[Title/Abstract] OR "median nerve compression"[Title/Abstract])

AND

("recurrent"[Title/Abstract] OR "recurrence"[Title/Abstract] OR "revision"[Title/Abstract] OR "reoperation"[Title/Abstract]

OR "repeat"[Title/Abstract])

AND

("surgery"[Title/Abstract] OR "surgical treatment"[Title/Abstract] OR "neurolysis"[Title/Abstract]

OR "decompression"[Title/Abstract] OR "flap"[Title/Abstract] OR "fat graft"[Title/Abstract]

OR "hypothenar fat pad"[Title/Abstract] OR "synovial wrap"[Title/Abstract]

OR "collagen wrap"[Title/Abstract] OR "umbilical cord allograft"[Title/Abstract]

OR "porcine extracellular matrix"[Title/Abstract] OR "external neurolysis"[Title/Abstract]

OR "nerve coverage"[Title/Abstract] OR "nerve wrap"[Title/Abstract] OR "revision carpal tunnel release"[Title/Abstract]

OR "endoscopic carpal tunnel release"[Title/Abstract]))
